# Mechanical Identification of Materials and Structures with Optical Methods and Metaheuristic Optimization

**DOI:** 10.3390/ma12132133

**Published:** 2019-07-02

**Authors:** Elisa Ficarella, Luciano Lamberti, Sadik Ozgur Degertekin

**Affiliations:** 1 Dipartimento di Meccanica, Matematica e Management, Politecnico di Bari, 70126 Bari, Italy; 2 Department of Civil Engineering, Dicle University, 21280 Diyarbakır, Turkey

**Keywords:** optical methods, inverse problems, hybrid metaheuristic algorithms, simulated annealing, harmony search, big bang-big crunch

## Abstract

This study presents a hybrid framework for mechanical identification of materials and structures. The inverse problem is solved by combining experimental measurements performed by optical methods and non-linear optimization using metaheuristic algorithms. In particular, we develop three advanced formulations of Simulated Annealing (SA), Harmony Search (HS) and Big Bang-Big Crunch (BBBC) including enhanced approximate line search and computationally cheap gradient evaluation strategies. The rationale behind the new algorithms—denoted as Hybrid Fast Simulated Annealing (HFSA), Hybrid Fast Harmony Search (HFHS) and Hybrid Fast Big Bang-Big Crunch (HFBBBC)—is to generate high quality trial designs lying on a properly selected set of descent directions. Besides hybridizing SA/HS/BBBC metaheuristic search engines with gradient information and approximate line search, HS and BBBC are also hybridized with an enhanced 1-D probabilistic search derived from SA. The results obtained in three inverse problems regarding composite and transversely isotropic hyperelastic materials/structures with up to 17 unknown properties clearly demonstrate the validity of the proposed approach, which allows to significantly reduce the number of structural analyses with respect to previous SA/HS/BBBC formulations and improves robustness of metaheuristic search engines.

## 1. Introduction and Theoretical Background

An important type of inverse problems is to identify material properties involved in constitutive equations or stiffness properties that drive the mechanical response to applied loads. Since displacements represent the direct solution of the general mechanics problem for a body subject to some loads and kinematic constraints, the inverse solution of the problem is to identify structural properties corresponding to a given displacement field {*u*(x, y, z), *v*(x, y, z), *w*(x, y, z)}. The term “structural properties” covers material parameters (e.g., Young’s modulus, hyperelastic constants, viscosity etc.) and stiffness terms including details on material constituents (e.g., tension/shear/bending terms in composite laminates, fiber orientation and ply thickness etc.) evaluated for the region of the body under investigation.

The finite element model updating technique (FEMU) [1,2,3] and the virtual fields method (VFM) [2,3,4] are the most common approaches adopted in the literature for solving mechanical characterization problems. In general, FEMU is computationally more expensive than VFM but the latter method may require special cares in selecting specimen shape, virtual displacement fields and kinematic boundary conditions to simplify computations entailed by the identification process and obtain realistic results.

In the FEMU method, experimentally measured displacement fields are compared with their counterparts predicted by a finite element model simulating the experiment. This comparison is made at a given set of control points. If boundary conditions and loads are properly simulated by the FE model, computed displacements match experimental data only when the actual structural properties are given in input to the numerical model. The difference between computed displacement values and measured target values may be expressed as an error functional Ω that depends on the unknown material/structural properties to be identified. Hence, the inverse problem of identifying NMP unknown mechanical properties may be stated as an optimization problem where the goal is to minimize the error functional Ω. That is:

(1) $$\{\begin{array}{l} \mathrm{Min}\;\left[\Omega \left(X_{1},X_{2},\ldots ,X_{\mathrm{NMP}}\right)=\sqrt{\frac{1}{N_{\mathrm{CNT}}}\overset{N_{\mathrm{CNT}}}{\underset{j=1}{\sum }}{\left(\frac{\delta_{\mathrm{FEM}}{}^{j}-\bar{\delta^{j}}}{\bar{\delta^{j}}}\right)}^{2}}\right]\\ G_{p}\left(X_{1},X_{2},\ldots ,X_{\mathrm{NMP}}\right)\geq 0\\ X_{i}{}^{L}\leq X_{i}\leq X_{i}{}^{U} \end{array}\;\{\begin{array}{l} i=1,\ldots ,NMP\\ p=1,\ldots ,NC \end{array}$$

where: X_PROP_ (X_1_,X_2_,…,X_NMP_) is the design vector containing the NMP unknown properties ranging between the lower bounds “L” and the upper bounds “U”; N_CNT_ is the number of control points at which FE results are compared with experimental data; $\delta_{\mathrm{FEM}}{}^{j}$ and $\bar{\delta^{j}}$, respectively, are the computed and target displacement values at the j^th^ control point; G_p_(X_PROP_) define a set of NC constraint functions depending on unknown properties that must be satisfied in order to guarantee the existence of a solution for the inverse problem. Buckling loads or natural frequencies can also be taken as target quantities in the optimization process: in this case, the displacement field of the structure is described by the corresponding normalized mode shape.

Non-contact optical techniques [5,6,7] such as moiré, holography, speckle and digital image correlation are naturally suited for material/structure identification because they can accurately measure displacements in real time and gather full field information without altering specimen conditions. The full field ability of optical techniques allows to select the necessary amount of experimental data for making the results of identification process reliable. Furthermore, their versatility also allows to choose the best experimental set-up for the inverse problem at hand. Based on the type of illumination varying from coherent or partially coherent light to white light, the magnitude of measured displacements may range from fraction of microns (using, for example, lasers and interferometry) to some millimetres (using, for example, grating projection, image correlation and white light), thus covering a wide spectrum of materials and structural identification problems.

Regardless of the way displacement information are extracted from recorded images, all optical methods share a common basic principle. The light wave fronts hitting the specimen surface are modulated by the deformations undergone by the tested body. By comparing the wave fronts modulated by the body surface and recorded by a sensor before and after deformation a system of fringes forms on the specimen surface; each fringe represents the locus of an iso-displacement region. The spatial frequency distribution of fringes can be used for recovering strain fields. Material anisotropy, presence of local defects (e.g., dislocations in crystalline structures) and/or damage (e.g., delamination or cracks) produce fringe distortions or changes in spatial frequency of fringe patterns.

The inverse problem (1) is in general highly nonlinear and in all likelihood non-convex, especially if there are many parameters to be identified. Furthermore, the error functional Ω is not explicitly defined and each new evaluation of Ω entails a new finite element analysis. Such a non-smooth optimization problem cannot be handled efficiently by gradient-based algorithms. In fact, their utilization has continuously been decreasing in the last 10 years. For example, in the case of soft materials (a rather complicated subject) just a few studies using Levenberg-Marquardt or Sequential Quadratic Programming techniques (see, for example, [8,9,10,11,12,13,14,15,16]) have earned at least one or two citations per year.

Global optimization methods can explore larger fractions of design space than gradient-based algorithms. This results in better trial solutions and higher probability of avoiding premature convergence to local minima. A purely random search allows in principle to explore the whole design space but it may be computationally unaffordable because the number of trial solutions yielding reductions of Ω rapidly decreases as the optimization process progresses. In order to rationalize search process and improve computational speed of global optimization, metaheuristic algorithms have been developed inspired by evolution theory, medicine, biology and zoology, physics and astronomy, human sciences etc. Trial designs are randomly generated according to the selected inspiring principle. Metaheuristic methods have been successfully utilized practically in every field of science and engineering.

Genetic algorithms (GA) [17,18], evolution strategies (ES) [19,20,21] and simulated annealing (SA) [22,23] were among the first metaheuristic optimization methods to be developed in the early ‘1980s and are still widely utilized nowadays. The basic difference between GA/ES and SA is that the former algorithms operate with a population of candidate designs while the latter algorithm, at least in its classical implementation, considers one trial design at a time and then further develops it.

Swarm intelligence algorithms mostly inspired by animals’ behavior are other population-based algorithms developed since early 1990s. They still attract the attention of optimization experts that continue to propose new algorithms: the most popular methods are particle swarm optimization (PSO) [24], ant colony optimization (ACO) [25], artificial bee colony (ABC) [26], firefly algorithm (FFA) [27], bat algorithm (BA) [28], and cuckoo search (CS) [29].

Social sciences and human activities have been for almost 20 years another important source of inspiration for metaheuristic algorithms, yet they not as popular as swarm intelligence methods. Among others, we can mention tabu search (TS) [30], harmony search (HS) [31], imperialist competitive algorithm (ICA) [32], teaching-learning based optimization (TLBO) [33], search group algorithm (SGA) [34], and JAYA [35].

Astronomy, physics (electromagnetism, optics, classical mechanics, etc.) and natural phenomena have provided another prolific field of inspiration, especially in the last 10–15 years: for example, big bang-big crunch (BBBC) [36], gravitational search algorithm (GSA) [37], charged system search (CSS) [38], colliding bodies optimization (CBO) [39], ray optimization [40], water evaporation optimization (WEO) [41], thermal exchange optimization (TEO) [42], and cyclical parthenogenesis algorithm (CPA) [43], just to mention a few.

A rapid survey of the optimization literature produced over the last 15 years reveals that GA [44,45,46,47,48,49,50,51,52,53,54,55,56,57,58,59,60,61,62], DE [63,64,65,66,67,68,69,70,71,72,73,74,75], SA [76,77,78,79,80,81,82,83,84,85,86,87,88,89,90,91,92,93,94,95,96,97], HS [98,99,100,101,102,103,104,105,106,107,108,109,110,111,112] and PSO [113,114,115,116,117,118,119,120,121,122,123,124,125,126,127,128,129,130,131,132,133] are the most popular metaheuristic algorithms used in mechanical identification problems. In order to improve computational efficiency of identification process, GA and SA were often hybridized [134,135,136,137]. Similarly, PSO was hybridized with many other algorithms including, for example, GA [138,139], GA and ACO [140] and other swarm intelligence methods [141]. HS was hybridized with GA [142] and PSO/RO [143].

BBBC [144,145,146,147,148,149] was more often utilized than ICA [150,151], ACO [152,153], JAYA [154,155] and machine learning [156]. However, there are quite less studies on inverse problems employing BBBC than for GA, DE, SA, HS and PSO. Such a difference may be explained with the informal argument that BBBC was developed much later than GA, DE, SA, HS and PSO.

The many studies listed above is a direct consequence of the blooming of metaheuristic methods favored by the exponentially increasing computational power. Applications of metaheuristic algorithms to inverse problems with special emphasis on material characterization and structural damage detection are critically reviewed in [157,158,159,160,161]. From the stand point of algorithmic formulation, it should be noted that SA is the only metaheuristic algorithm inherently capable of bypassing local optima. However, HS and BBBC include very important features that should be possessed by any population-based algorithm. In particular, HS stores all candidate designs (i.e., those forming the population and additional designs kept in memory from previous iterations) in a matrix called harmony memory. Values assigned to optimization variables can be extracted from this memory to form new trial designs. This allows one to carry out an adaptive search while avoiding stagnation. BBBC utilizes the concept of center of mass, which makes it possible to follow the evolution of the average characteristics of the population over the optimization process. GA and PSO instead may suffer from premature convergence, stagnation, sensitivity to problem formulation. Furthermore, GA and PSO include more internal parameters than SA, HS and BBBC, which increases the amount of heuristics in the optimization process. The same arguments may be used for DE whose performance is strongly dependent on the crossover/mutation scheme implemented in the algorithm. Based on these considerations, we decided to develop advanced formulations of SA, HS and BBBC for material/structural identification problems.

Lamberti et al. attempted to improve the convergence speed of SA (e.g., [77,81,83,84,162,163]), HS (e.g., [164,165,166]) and BBBC (e.g., [165,166]) in inverse and structural optimization problems. While these SA/HS/BBBC variants clearly outperformed referenced algorithms in weight minimization of skeletal structures, improvements in computational cost were less significant for inverse problems as those variants evaluated gradients of error functional Ω using a “brute-force” approach based on finite differences. This occurred in spite of having enriched metaheuristic search with gradient information. In order to overcome this limitation, this study presents new hybrid formulations of SA, HS and BBBC that are significantly more efficient and robust than the algorithms currently available in the literature. For that purpose, low-cost gradient evaluation and approximate line search strategies are introduced in order to generate higher quality trial designs and a very large number of descent directions. Populations of candidate designs are renewed very dynamically by replacing the largest number of designs as possible. All algorithms use a very fast 1-D probabilistic search derived from simulated annealing.

The new algorithms—denoted as Hybrid Fast Simulated Annealing (HFSA), Hybrid Fast Harmony Search (HFHS) and Hybrid Fast Big Bang-Big Crunch (HFBBBC)—are tested in three inverse elasticity problems: (i) mechanical characterization of a composite laminate used as substrate in electronic boards (four unknown elastic constants); (ii) mechanical characterization and layup identification of a composite unstiffened panel for aeronautical use (four unknown elastic constants and three unknown layup angles); (iii) mechanical characterization of bovine pericardium patches used in biomedical applications (sixteen unknown hyperelastic constants and the fiber orientation). Sensitivity of inverse problem solutions and convergence behavior to population size and initial design/population is evaluated in statistical terms.

The rest of this article is structured as follows: Section 2, Section 3 and Section 4, respectively, describe the new SA, HS and BBBC formulations developed here trying to point out the theoretical aspects behind the proposed enhancements and critically compare the new formulations with currently available SA/HS/BBBC variants including those developed in [77,81,83,84,162,166]. Section 5 presents the results obtained in the inverse problems. Finally, Section 6 discusses the main findings of this study.

## 2. Hybrid Fast Simulated Annealing

**Figure 1:**
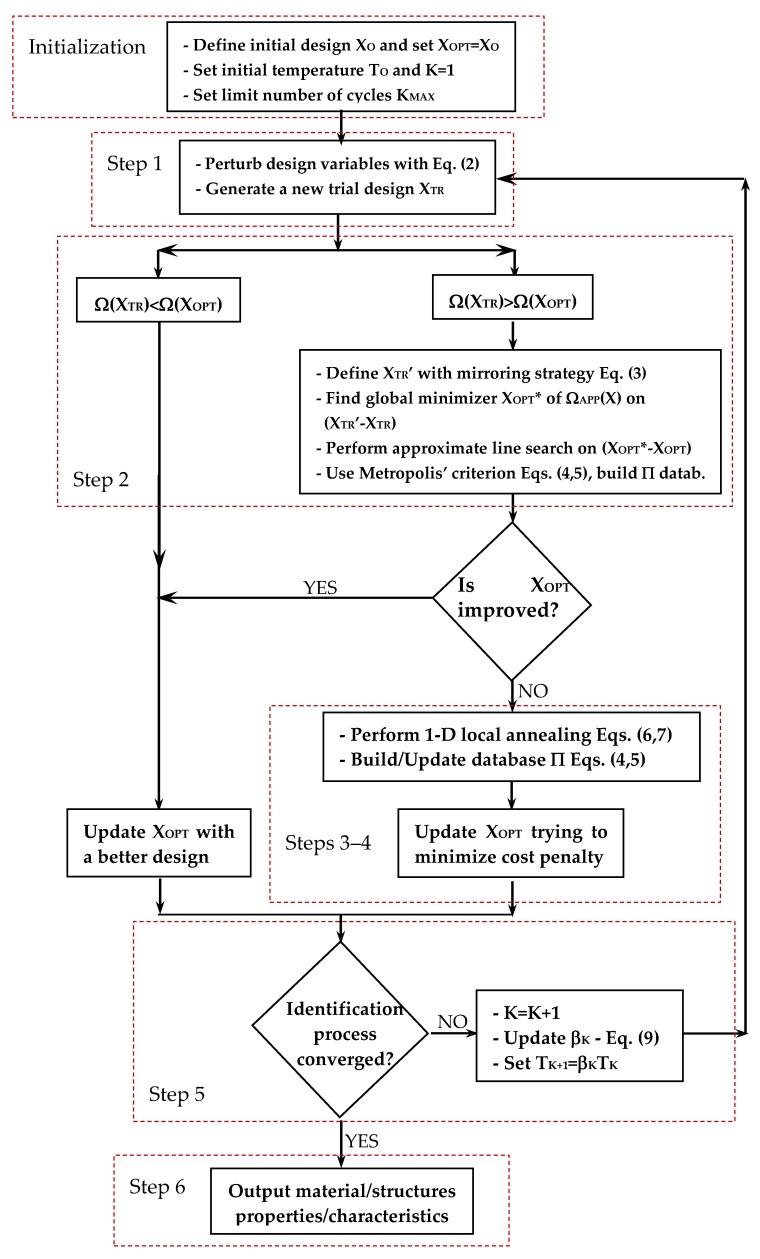
Flow chart of the HFSA algorithm developed in this research.

The flow chart of the new HFSA algorithm developed in this study is shown in Figure 1. HFSA includes a multi-level and multi-point formulation combining global and local annealing, evaluation of multiple trial points, and line search strategies based on fast gradient computation. The hybrid nature of HFSA derives from the fact that metaheuristic search is enriched by approximate line searches. Similar to classical SA, the proposed algorithm starts with setting an initial design vector **X_0_** as the current best record **X_OPT_**. The corresponding cost function value Ω_OPT_ = Ω(**X_OPT_**) is computed. Set the counter of cooling cycles as K = 1 and the maximum number of cooling cycles as K_MAX_ = 100. Set the initial temperature T_0_ equal to 0.1 near to the target value 0 of the error functional Ω.

### 2.1. Step 1: Generate A New Trial Design with “Global” Annealing by Perturbing All Design Variables

Since determination of sensitivities ∂Ω/∂x_j_ entails new structural analyses, material parameters taken as optimization variables are perturbed as follows:

(2) $$x_{j}\;=\;x_{\mathrm{OPT},j}\;-\;\left(x_{\mathrm{OPT},j}{}^{l}-x_{\mathrm{OPT},j}{}^{l-1}\right)\frac{\left|\Omega_{\mathrm{OPT}}{}^{l}-\Omega_{\mathrm{OPT}}{}^{l-1}\right|}{\left\|X_{\mathrm{OPT}}{}^{l}-X_{\mathrm{OPT}}{}^{l-1}\right\|}\times N_{\mathrm{RND},j}\;\times \;{Ω}_{\mathrm{OPT},l-1}/{Ω}_{\mathrm{OPT},l}\;\;(j\;=\;1,\ldots ,\mathrm{NMP})$$

where **X**_OPT_*^l^* and **X**_OPT_*^l^*^−1^ are the best records for the last two iterations; (**X**_OPT_*^l^*) and (**X**_OPT_*^l^*^−1^) are the corresponding values of error functional; N_RND,j_ is a random number in the interval (0,1).

If Ω(**X**_OPT_*^l^*) < Ω(**X**_OPT_*^l^*^−1^), (**X**_OPT_*^l^* − **X**_OPT_*^l^*^−1^) is a descent direction with respect to the previous best record **X**_OPT_*^l^*^−1^ while −(**X**_OPT_*^l^* − **X**_OPT_*^l^*^−1^) may be a descent direction with respect to the current best record **X**_OPT_*^l^*. The approximate gradient of Ω is computed as |Ω_OPT_*^l^* − Ω_OPT_*^l^*^−1^|/||**X**_OPT_*^l^* − **X**_OPT_*^l^*^−1^||: the absolute value accounts for the “−” sign included in Equation (2). The N_RND,j_ random number preserves the heuristic character of the SA search while the Ω_OPT,*l*−1_/Ω_OPT,*l*_ ratio forces the optimizer to take a large step along a potentially descent direction. Using approximate gradient evaluation allows computational cost of the inverse problem to be drastically reduced with respect to other SA applications [76,77,81,83,84].

A trial design **X_TR_**(x_OPT,1_ + Δx_1_, x_OPT,2_ + Δx_2,…,_x_OPT,NMP−1_ + Δx_NMP−1_, x_OPT,NMP_ + Δx_NMP_) is hence formed.

### 2.2. Step 2: Evaluation of the New Trial Design

If Ω(X_TR_) < Ω(X_OPT_), X_TR_ is set as the new best record X_OPT_. Step 5 is executed in order to check for convergence and reset parameters K and T_K_.

If Ω(X_TR_) > Ω(X_OPT_), a “mirroring strategy” is used to perturb design along a descent direction. In fact, since Ω(X_TR_) > Ω(X_OPT_) yields (X_TR_ − X_OPT_)^T^$\bar{\nabla }$Ω(X_OPT_) > 0, the −(X_TR_ − X_OPT_)^T^$\bar{\nabla }$Ω(X_OPT_) < 0 condition is expected to be satisfied thus defining the new descent direction (X_TR_^new^ − X_OPT_)≡−(X_TR_ − X_OPT_). The new candidate design X_TR_^new^ is defined as:

**X_TR_^new^** = **2X_OPT_** − **X_TR_** (3)

If the mirror trial point **X_TR_^new^** yet does not improve **X_OPT_** (i.e., if Ω(**X_TR_^new^**) > Ω(**X_OPT_**)), the cost function Ω(**X**) is approximated by a 4th order polynomial that passes through the five trial points **X_TR_**, **X_INT_^’^**, **X_OPT_**, **X_INT_^’’^** and **X_TR_^new^** where **X_INT_^’^** is randomly generated on the segment limited by **X_TR_** and **X_OPT_** while **X_INT_^’’^** is randomly generated on the segment limited by **X_OPT_** and **X_TR_^new^**. A local 1-D coordinate system is set for the segment limited by **X_TR_** and **X_TR_^new^**: the origin is located at **X_OPT_** and coordinates are normalized with respect to the distance from the origin. The trial point X_OPT_* at which the approximate error functional Ω_APP_(**X**) takes its minimum value is determined. An exact analysis is performed at X_OPT_* and the real value of error functional Ω(X_OPT_*) is computed. The following cases may occur.

If Ω(X_OPT_*) < Ω(**X_OPT_**), X_OPT_* is reset as the current best record. Hence, Step 5 is executed in order to check for convergence and reset K and T_K_.

If Ω(X_OPT_*) > Ω(**X_OPT_**), trial designs **X_TR_**, **X_INT_^’^**, X_OPT_*, **X_INT_^’’^** and **X_TR_^new^** are evaluated with the Metropolis’ criterion. The cost function variation ΔΩ_s_ = [Ω(**X_s_**) − Ω(**X_OPT_**)] is computed for these designs (the s subscript denotes TR, INT’, OPT*, INT” and TR^new^, respectively). For the trial design yielding the smallest increment ΔΩ_s_ > 0 (in all likelihood X_OPT_*), the Metropolis’ probability function is defined as:

(4) $$P(\Delta {Ω}_{s})=\;e^{\frac{-{\Delta \Omega }_{s}}{\left(\sum_{r=1}^{\mathrm{NDW}}{\Delta \Omega }_{r}/\mathrm{NDW}\right)\cdot T_{K}}}$$

where NDW are the trial points at which error functional was higher than the previously found best records up the current iteration. The ΔΩ_r_ terms are the corresponding cost penalties. The ratio ∑_r=1, NDW_ ΔΩ_r_/NDW accounts for the general formation of all previous trial designs and normalizes probability function with respect to cost function changes.

The design **X**_s_ is provisionally accepted or certainly rejected according to the Metropolis’ criterion:
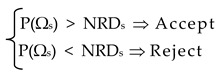
(5)
where NRD_s_ is a random number defined in the interval (0, 1).

If **X**_s_ may be accepted from Equation (5), it is added to the database Π, that includes all trial designs that could not improve the current best record. Hence, Step 4 is executed.

If all of the **X**_s_ points are rejected from Equation (5), Step 3 is executed.

### 2.3. Step 3: Generate New Designs with “Local” Annealing by Perturbing One Variable at A Time

In classical SA, M_ann_ cycles are completed and a total of M_ann_·NMP analyses are performed. Here, derivatives ∂Ω/∂x_j_ (j = 1,2,…,NMP) computed at **X_OPT_** are sorted in ascending order from the minimum value to the maximum value. Following this order, design variables are perturbed one by one as:

x_j_^TR,1d^ = x_OPT,j_ ± (x_j_^U^ − x_j_^L^)·N_RND,j_   (j = 1,2,…,NMP) (6)

where the sign “+” is used if ∂Ω/∂x_j_ < 0 while the sign “−” is used if ∂Ω/∂x_j_ > 0. If x_j_^TR^ violates side constraints, it is reset as follows:
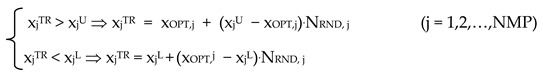
(7)

Each new design **X_TR_^j^**(x_OPT,1_,x_OPT,2_,…,x_j_^TR^,…,x_OPT,NMP−1_,x_OPT,NMP_) defined with Equations (6) and (7) is evaluated and the current best record is updated if it holds Ω(**X_TR_^j^**) < Ω(**X_OPT_**). Conversely, if Ω(**X_TR_^j^**) > Ω(**X_OPT_**), the mirror trial point **X_TR_^j,mirr^**(x_OPT,1_,x_OPT,2_,2x_OPT_−x_j_^TR^,…,x_OPT,NMP−1_,x_OPT,NMP_) is evaluated. Two scenarios may occur: (i) if Ω(**X_TR_^j,mirr^**) < Ω(**X_OPT_**), **X_TR_^j,mirr^** is set as the new best record **X_OPT_**; (ii) if also Ω(**X_TR_^j,mirr^**) > Ω(**X_OPT_**), **X_TR_^j^** and **X_TR_^j,mirr^** are evaluated with the Metropolis criterion (5) and the best of them is eventually set as the current best record. The 1-D search lasts until no improvement in design is achieved over two consecutive cycles.

Similar to the “global” annealing strategy, the 1-D probabilistic search attempts to generate trial designs lying on descent directions. However, perturbation initiates from the most sensitive variables in order to capture the effect of each single variable in a more efficient way. In fact, in the global annealing search, the vector (**X_TR_** − **X_OPT_**) was defined so as to have (**X_TR_** − **X_OPT_**)^T^$\bar{\nabla }$Ω(**X_OPT_**) < 0, thus forming a descent direction. However, non-linearity of cost function made such a condition be not sufficient for improving design. In view of this, “local” annealing selects the most important terms (**X_TR_** − **X_OPT_**)^j^∂Ω/∂x_j_ that form the cost function variation and promptly correct them should they not contribute effectively to the reduction of cost function.

### 2.4. Step 4: Evaluation of Trial Designs that Satisfy Metropolis’ Criterion

If there are no trial designs for which the cost function decreases, HFSA extracts from the database Π (including designs that satisfy the Metropolis’ criterion) the design **X**_j_^BEST^ for which the cost function value is the least, and then sets this design as the current best record. Hence, the increase in cost is minimized each time all improvement routines failed and the 1-D local annealing search could not improve design.

### 2.5. Step 5: Check for Convergence and Eventually Reset Parameters for A New Cooling Cycle

If the annealing cycles counter K > 3, HFSA utilizes the following convergence criterion:

(8) $$\mathrm{Max}\{\mathrm{Max}[\begin{array}{c} \frac{\left|\Omega_{\mathrm{OPT},K}-\Omega_{\mathrm{OPT},K-1}\right|}{\Omega_{\mathrm{OPT},K}};\\ \frac{\left|\Omega_{\mathrm{OPT},K-1}-\Omega_{\mathrm{OPT},K-2}\right|}{\Omega_{\mathrm{OPT},K-1}};\\ \frac{\left|\Omega_{\mathrm{OPT},K-2}-\Omega_{\mathrm{OPT},K-3}\right|}{\Omega_{\mathrm{OPT},K-2}} \end{array}];\mathrm{Max}[\begin{array}{c} \frac{\left|\left|X_{\mathrm{OPT},K}-X_{\mathrm{OPT},K-1}\right|\right|}{\left|\left|X_{\mathrm{OPT},K}\right|\right|};\\ \frac{\left|\left|X_{\mathrm{OPT},K-1}-X_{\mathrm{OPT},K-2}\right|\right|}{\left|\left|X_{\mathrm{OPT},K-1}\right|\right|};\\ \frac{\left|\left|X_{\mathrm{OPT},K-2}-X_{\mathrm{OPT},K-3}\right|\right|}{\left|\left|X_{\mathrm{OPT},K-2}\right|\right|} \end{array}]\}\leq \varepsilon_{\mathrm{CONV}}$$

where Ω_OPT,K_ and **X**_OPT,K,_ respectively, are the best record and corresponding design vector obtained in the K^th^ cooling cycle. The convergence parameter ε_CONV_ is set equal to 10^−7^.

If the criterion (8) is satisfied or K = K_MAX_, go to Step 6.

Conversely, if K < 3 or stopping criterion is not satisfied (also for K ≥ 3), the number of cooling cycles is reset as K = K + 1. The temperature is adaptively reduced as T_K+1_ = β_K_ T_K_ where:

(9) $$\beta_{K}=\left[\overset{K-1}{\underset{r=0}{\sum }}\beta_{r}/K\right]\;\times \;\mathrm{Max}\left[0.95/\left(1+\frac{N_{\mathrm{REJE}}}{N_{\mathrm{TRIA}}}\right);\left(1-\frac{\Omega_{\mathrm{FIN},K-1}}{\Omega_{\mathrm{INIT},K-1}}\right)\right]$$

Ω_INIT,K−1_ and Ω_FIN,K−1_, respectively, are the cost function values at the beginning and at the end of current annealing cycle. N_REJE_ is the number of trial designs rejected out of total number of trial designs N_TRIA_ generated in the current cooling cycle.

### 2.6. Step 6: End Optimization Process

HFSA terminates the optimization process and writes the output data in the results file.

## 3. Hybrid Fast Harmony Search

**Figure 2:**
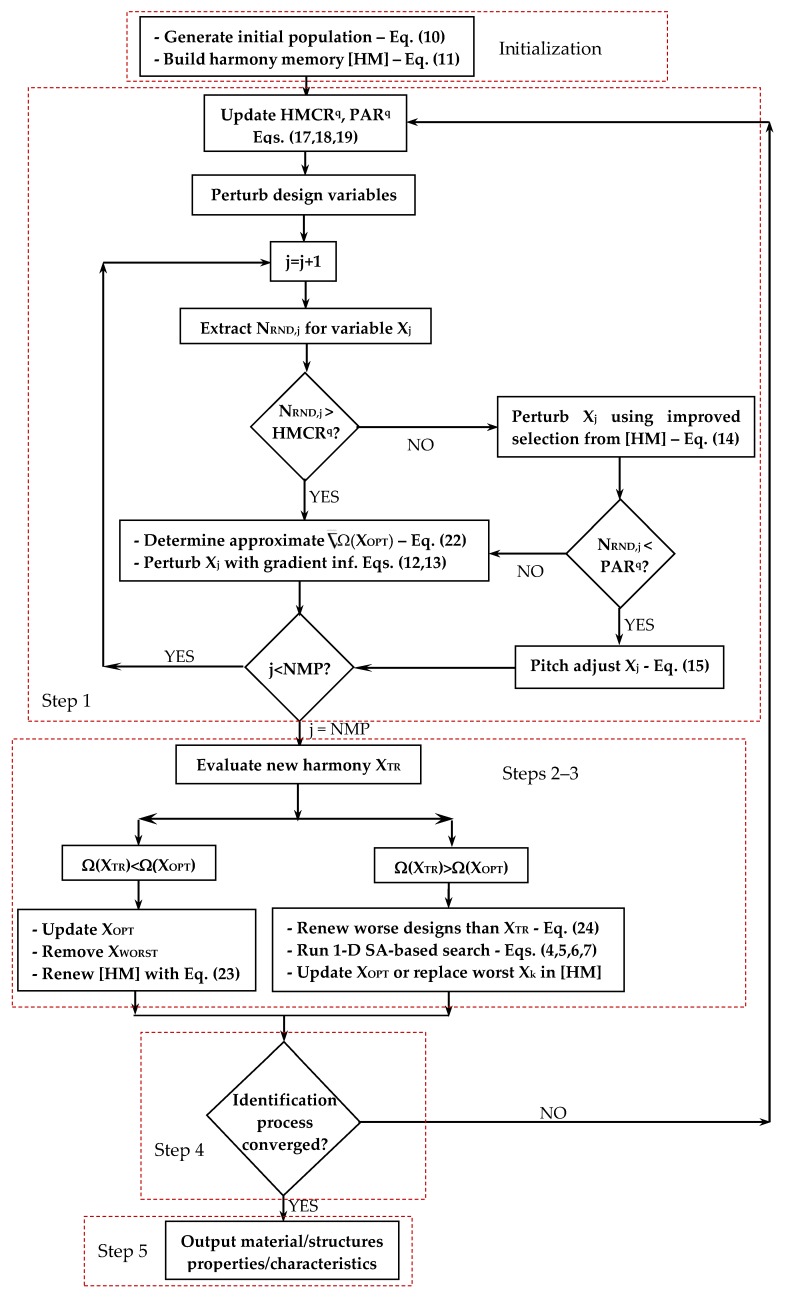
Flow chart of the HFHS algorithm developed in this research.

The new HFHS algorithm developed in this research is now described in detail. Like HFSA, HFHS enriches the metaheuristic search with gradient information and approximate line searches. This is done at low computational cost. Furthermore, the HS engine is enhanced by a 1-D probabilistic search based on simulated annealing. Like many state-of-the-art HS variants, internal parameters such as harmony memory considering rate (HMCR) and pitch adjusting rate (PAR) are adaptively changed by HFHS in the optimization process based on convergence history. Last, classical harmony refinement process based on bandwidth parameter (bw) is replaced by another random movement that forces HFHS to refine the new harmony moving along a descent direction. The flow chart of the new algorithm is presented in Figure 2.

The initial population of N_POP_ solutions is randomly generated using the following equation:

(10) $$x_{j}^{k}=x_{j}^{L}+\rho_{j}{}^{k}\;(x_{j}^{U}-x_{j}^{L})\;\;\;(k\;=\;1,2,\ldots N_{\mathrm{POP}};\;j\;=\;1,2,\ldots ,\mathrm{NMP})$$

where ρ_j_^k^ is a random number uniformly generated in the (0,1) interval.

These designs are sorted in ascending order according to values taken by the error functional Ω.

(11) $$\left[\mathrm{HM}\right]=[\begin{array}{l} x_{1}^{1}\\ x_{2}^{1}\\ \ldots \\ x_{1}^{N_{\mathrm{POP}}-1}\\ x_{1}^{N_{\mathrm{POP}}} \end{array}\begin{array}{l} x_{2}^{1}\\ x_{2}^{2}\\ \ldots \\ x_{2}^{N_{\mathrm{POP}}-1}\\ x_{2}^{N_{\mathrm{POP}}} \end{array}\begin{array}{l} \ldots \\ \ldots \\ \ldots \\ \ldots \\ \ldots \end{array}\begin{array}{l} x_{\mathrm{NMP}-1}^{1}\\ x_{\mathrm{NMP}-1}^{2}\\ \ldots \\ x_{\mathrm{NMP}-1}^{N_{\mathrm{POP}}-1}\\ x_{\mathrm{NMP}-1}^{N_{\mathrm{POP}}} \end{array}\begin{array}{l} x_{\mathrm{NMP}}^{1}\\ x_{\mathrm{NMP}}^{2}\\ \ldots \\ x_{\mathrm{NMP}}^{N_{\mathrm{POP}}-1}\\ x_{\mathrm{NMP}}^{N_{\mathrm{POP}}} \end{array}]$$

As mentioned above, the present HFHS algorithm does not require initialization of internal parameters HMCR, PAR and bw.

### 3.1. Step 1: Generation and Adjustment of a New Harmony with Adaptive Parameter Selection

Let **X_OPT_** = {x_OPT,1_,x_OPT,2_,…,x_OPT,NMP_} be the best design stored in the population corresponding to Ω**_OPT_**. The gradient of error functional with respect to design variables $\bar{\nabla }$Ω(**X_OPT_**) is computed at **X_OPT_**. For each variable, a random number N_RND,j_ is extracted from the (0,1) interval.

If N_RND,j_ > HMCR, the new value x_TR,j_ assigned to the j^th^ optimization variable (j = 1,2,…,NMP) currently perturbed is:
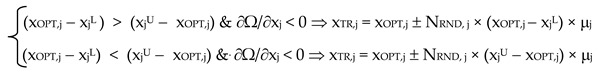
(12)
where ∂Ω/∂x_j_ is the cost function sensitivity for the j^th^ design variable currently perturbed, μ_j_ = (∂Ω/∂x_j_)/||$\bar{\nabla }$Ω(**X_OPT_**)|| is the sensitivity coefficient normalized with respect to the gradient vector modulus. Sensitivities ∂Ω/∂x_j_ are computed with Equation (22), which will be described later on in this section. Using the ‘+’ sign if it holds ∂Ω/∂x_j_ < 0 and the ‘−’ sign if it holds ∂Ω/∂x_j_ > 0, allows to generate trial points lying on descent directions.

If HMCR is small, Equation (12) is more likely to be used. The (x_TR,j_ − x_OPT,j_) perturbations given to each design variable are weighted by sensitivities to form the cost function variation ΔΩ_TR_ for the new harmony **X_TR_**. This variation is expressed by the scalar product ΔΩ_TR_ between the gradient $\bar{\nabla }$Ω(**X_OPT_**) and the search direction **S_TR_^T^** = (**X_TR_** − **X_OPT_**) formed by the new harmony and the current best record. If ΔΩ_TR_ < 0, **S_TR_^T^** is a descent direction. In order to make **S_TR_^T^** a descent direction, all increments (x_TR,j_ − x_OPT,j_) ∂Ω/∂x_j_ must hence be negative. The following strategy is adopted to retain or adjust the perturbation given to the current design variable (j = 1,2,…,NMP):
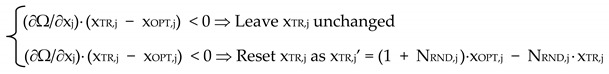
(13)

Hence, Equations (12) and (13) randomly generate new trial designs that must lie on descent directions. Sensitivities are computed at the current best record to improve convergence speed. The mirroring strategy implemented by the second relationship of Equation (13) attempts to transform the non-descent direction **S_TR_** into the descent direction −**S_TR_** by perturbing design in the opposite direction.

The effect of the distance of current best record **X_OPT_** from side constraint boundaries is taken into account by perturbing design variables by the largest step as possible (i.e., (x_OPT,j_ − x_j_^L^) or (x_j_^U^ − x_OPT,j_)) along the currently defined descent direction. This allows to maximize the improvement in cost function.

If N_RND,j_ < HMCR, the new value x_TR,j_ assigned to the j^th^ variable is defined as (j = 1,2,…,NMP):

(14) $$x_{TR,j}={\hat{x}}_{TR,j}^{HM}+\left(N_{RND,j}-0.5\right)\times Max\left[\left({\hat{x}}_{TR,j}^{HM}-{\hat{x}}_{TR,j}^{HM,less}\right),\left({\hat{x}}_{TR,j}^{HM,more}-{\hat{x}}_{TR,j}^{HM}\right)\right]$$

where ${\hat{x}}_{\mathrm{TR},j}^{\mathrm{HM},\mathrm{less}}$ and ${\hat{x}}_{\mathrm{TR},j}^{\mathrm{HM},\mathrm{more}}$ are two adjacent values to the ${\hat{x}}_{\mathrm{TR},j}^{\mathrm{HM}}$ value stored in [HM], such that ${\hat{x}}_{\mathrm{TR},j}^{\mathrm{HM},\mathrm{less}}$ < ${\hat{x}}_{\mathrm{TR},j}^{\mathrm{HM}}$ < ${\hat{x}}_{\mathrm{TR},j}^{\mathrm{HM},\mathrm{more}}$. Unlike classical HS and advanced formulations [163,164,167], HFHS does not select the value x_TR,j_ from the j^th^ column of the harmony memory storing values of the corresponding variable for each design of the population. This enhances diversity of optimization process and allows to avoid stagnation.

By considering the difference (N_RND,j_ − 0.5), it is possible to increase or reduce the ${\hat{x}}_{\mathrm{TR},j}^{\mathrm{HM}}$ value. The x_TR,j_ value is then adjusted with Equation (13) to make also step (x_TR,j_ − x_OPT,j_) lie on a descent direction. Conversely, in classical HS and [164,165,166], the pitch adjusting operation did not include any information on how much the design may be sensitive to the currently analyzed variable.

If it holds also N_RND,j_ < Min(HMCR,PAR), the x_TR,j_ value is finally pitch adjusted as:

(15) $$x_{TR,j}^{pitch,adj}=x_{TR,j}+\lambda_{scale}\times N_{RND,j}\times \frac{\left|x_{TR,j}-x_{OPT,j}\right|}{NG_{tot}}\times NG_{pitch,adj}\;(j\;=\;1,2,\ldots ,\mathrm{NMP})$$

where NG_pitch,adj_ is the number of previously pitch adjusted trial designs; NG_tot_ is the total number of trial designs generated in the optimization search. The NG_pitch,adj_ parameter is reset as (NG_pitch,adj_ + 1) if the number of pitch adjusted variables included in a new harmony is larger than the number of design variables perturbed with Equation (12) using gradient information.

The scale parameter λ_scale_ is set as:

(16) $$\lambda_{\mathrm{scale}}=\left\{ \begin{array}{l} \left(X_{\mathrm{TR},j}-x_{\mathrm{OPT},j}\right)<0\;\Rightarrow -1\\ \left(X_{\mathrm{TR},j}-x_{\mathrm{OPT},j}\right)>0\;\Rightarrow 1 \end{array}\right.\;\;\;(j\;=\;1,2,\ldots ,\mathrm{NMP})$$

Equations (14)–(16) replace the bandwidth parameter bw usually used in many HS variants. The new harmony **X_TR_**(x_TR,1_,x_TR,2_,…,x_TR,NMP_) can be decomposed in NMP sub-harmonies **X_TR,j_**(x_OPT,1_,x_OPT,2_, …,x_TR,j_,…,x_OPT,NMP_) obtained by perturbing only one design variable at a time, and that lie on descent directions. These movements are amplified by the scale factor defined by Equation (16). Furthermore, Equation (15) accounts also for optimization history. In fact, since NG_pitch,adj_/NG_tot_ decreases as optimization progresses, the perturbation step defined to pitch adjust each design variable gets finer as the optimum is approached.

The case N_RND,j_ < HMCR and N_RND,j_ > PAR is dealt with Equation (13) eventually including the mirroring strategy. Hence, the present algorithm intrinsically pitch adjusts design variables and tries anyhow to improve the current design.

As mentioned above, new values of HMCR and PAR parameters are randomly generated in each new iteration and adapted based on optimization history. In the q^th^ iteration, HMCR and PAR are set as:

(17) $${\mathrm{HMCR}}^{q}={\mathrm{HMCR}}_{\mathrm{extracted}}{}^{q}\times \frac{\Omega_{\mathrm{aver},\mathrm{end}}{}^{q-1}}{\Omega_{\mathrm{aver},\mathrm{init}}{}^{q-1}}\times \frac{{\mathrm{NG}}_{\mathrm{pitch},\mathrm{adj}}}{{\mathrm{NG}}_{\mathrm{gradient}}}$$

(18) $${\mathrm{PAR}}^{q}={\mathrm{PAR}}_{\mathrm{extracted}}{}^{q}\times \frac{\Omega_{\mathrm{aver},\mathrm{end}}{}^{q-1}}{\Omega_{\mathrm{aver},\mathrm{init}}{}^{q-1}}\times \frac{|\left|X_{\mathrm{OPT},\mathrm{end}}-X_{\mathrm{WORST},\mathrm{end}}\right|{|}^{q-1}}{|\left|X_{\mathrm{OPT},\mathrm{init}}-X_{\mathrm{WORST},\mathrm{init}}\right|{|}^{q-1}}\times \frac{{\mathrm{NG}}_{\mathrm{pitch},\mathrm{adj}}}{{\mathrm{NG}}_{\mathrm{gradient}}}$$

In Equations (17) and (18), Ω_aver,init_^q−1^ and Ω_aver,end_^q−1^, respectively, are the average values of cost function for the trial designs included in the harmony memory at the beginning and the end of the previous optimization iteration (the Ω_aver,end_^q−1^/Ω_aver,init_^q−1^ ratio should always be smaller than 1). **X_OPT,init_** and **X_WORST,init_**, **X_OPT,end_** and **X_WORST,end_**, respectively, denote the best and worst designs at the beginning and the end of the previous iteration. NG_gradient_ is the number of trial designs generated by including gradient information: this parameter is reset to (NG_gradient_ + 1) if the number of design variables perturbed with Equation (12) is greater than NMP/2.

Random values HMCR_extracted_^q^ and PAR_extracted_^q^ are defined as:

(19) $$\left\{ \begin{array}{l} HMCR_{extracted}{}^{q}=0.01+\xi_{HMCR}\times (0.99-0.01)\\ PAR_{extracted}{}^{q}=0.01+\xi_{PAR}\times (0.99-0.01) \end{array}\right.$$

where ξ_HMCR_ and ξ_PAR_ are two random numbers in the interval (0,1). The bounds of 0.01 and 0.99 set in Equation (19) allow all possible values of internal parameters to be covered [168].

In the first iteration (q = 1), it obviously holds HMCR^q^ = HMCR_extracted_^q^ and PAR^q^ = PAR_extracted_^q^.

Equations (17) and (18) rely on the following rationale: the error functional may decrease more rapidly if large perturbations are given to many variables. This is more likely to happen when gradient information is directly utilized, that is when it holds N_RND,j_ > HMCR. In order to increase the probability of using Equation (12) for many design variables, the HMCR value randomly generated is scaled by the (Ω_aver,end_^q−1^/Ω_aver,init_^q−1^) ratio. The generation process of new harmonies is hence forced to be consistent with the current rate of reduction of Ω.

Furthermore, HMCR^q^ is scaled by the NG_pitch,adj_/NG_gradient_ ratio. If the number of new harmonies generated via pitch adjusting tends to be smaller than the number of new harmonies directly generated including gradient information (i.e., if NG_pitch,adj_/NG_gradient_ < 1), it is more logical to keep following such a trend.

Similar arguments hold for the PAR^q^ parameter. Pitch adjustment is performed if N_RND,j_ < Min(HMCR,PAR). Besides information on cost function reduction rate (Ω_aver,end_^q−1^/Ω_aver,init_^q−1^), Equation (18) accounts for population diversity. In fact, pitch adjusting is less effective as population becomes less sparse, that is when the ||**X_OPT,end_** − **X_WORST,end_**||/||**X_OPT,init_** − **X_WORST,init_**|| ratio decreases. Again, the NG_pitch,adj_/NG_gradient_ ratio preserves the current trend of variation of the pitch adjusting rate parameter.

#### Determination of Sensitivities of Ω

Since the error functional is implicitly defined, gradients are determined by approximate line search. The cost function variation ΔΩ^k^ = [Ω(**X**^k^) − Ω(**X_OPT_**)] that occurs by moving from the best design **X_OPT_** to the k^th^ design **X**^k^ stored in the harmony memory is determined for all designs. The corresponding distance ΔS^k^ = ||**X**^k^−**X_OPT_**|| is computed. The approximate (i.e., “average”) gradient along each direction **S**^k^ = (**X**^k^ − **X_OPT_**) is computed as ΔΩ^k^/ΔS^k^. Since the new harmony must lie on a descent direction, the **S**^k^ vectors must be transformed into descent directions **S_desc_**^k^ = (**X_OPT_** − **X**^k^), that is **S_desc_**^k^ = −**ΔS**^k^. Three descent directions are considered: (i) best direction **S_BEST_** corresponding to the largest cost variation between candidate designs (this is the opposite direction to (**X_WORST_** − **X_OPT_**)); (ii) steepest descent direction **S_FAST_** corresponding to the largest gradient ΔΩ^k^/ΔS^k^; (iii) the second best direction **S_2ndBEST_** corresponding to the second largest cost variation between candidate designs (this is the opposite direction to (**X_2ndWORST_** − **X_OPT_**)).

**Figure 3:**
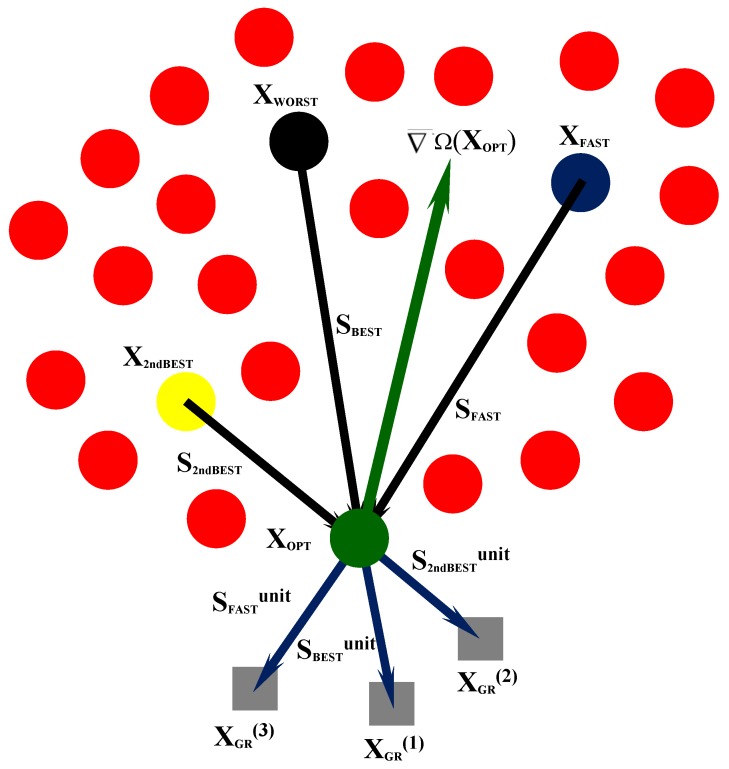
Determination of sensitivities for HFHS and HFBBBC. Red dots represent designs stored in the population. Since vectors (**X**_k_ − **X_OPT_**) are non-descent directions as (**X**_k_ − **X_OPT_**)^T^$\bar{\nabla }$Ω(**X_OPT_**) > 0, the opposite vectors are considered in order to perturb design along descent directions.

Figure 3 illustrates the formation of the descent directions and their mutual positions with respect to the gradient of cost functional. Any descent direction should be within the region limited by **S_BEST_**, **S_2ndBEST_** and **S_FAST_**. However, if the problem is highly nonlinear, it may happen that cost function oscillates along these directions and exceeds the current optimum cost. For this reason, step sizes are taken along **S_BEST_**, **S_2ndBEST_** and **S_FAST_**. The scale factors β_BEST_, β_2ndBEST_ and β_FAST_ are defined so that **S_BEST_^unit^**, **S_2ndBEST_^unit^** and **S_FAST_^unit^** are unit vectors. If ||**S_BEST_**|| < 1 or **||S_2ndBEST_**|| < 1 or **||S_FAST_**|| < 1, the corresponding unit direction coincides with the original direction and remains a descent direction. In order to check if unit directions are descent directions, three new trial designs **X_GR_**^(1)^, **X_GR_**^(2)^ and **X_GR_**^(3)^ are defined as:

(20) $$\left\{ \begin{array}{l} X_{\mathrm{GR}}{}^{\left(1\right)}=X_{\mathrm{OPT}}+S_{\mathrm{BEST}}{}^{\mathrm{unit}}\\ X_{\mathrm{GR}}{}^{\left(2\right)}=X_{\mathrm{OPT}}+S_{\text{2ndBEST}}{}^{\mathrm{unit}}\\ X_{\mathrm{GR}}{}^{\left(3\right)}=X_{\mathrm{OPT}}+S_{\mathrm{FAST}}{}^{\mathrm{unit}} \end{array}\right.$$

The **S_BEST_^unit^**, **S_2ndBEST_^unit^** and **S_FAST_^unit^** unit vectors are classified as descent directions if the following conditions hold true, respectively:

(21) $$\left\{ \begin{array}{l} \Omega \left(X_{\mathrm{GR}}{}^{\left(1\right)}\right)-\Omega_{\mathrm{OPT}}<0\Rightarrow S_{\mathrm{BEST}}{}^{unit}\;\;descent\\ \Omega \left(X_{\mathrm{GR}}{}^{\left(2\right)}\right)-\Omega_{\mathrm{OPT}}<0\Rightarrow S_{\text{2ndBEST}}{}^{unit}\;\;descent\\ \Omega \left(X_{\mathrm{GR}}{}^{\left(3\right)}\right)-\Omega_{\mathrm{OPT}}<0\Rightarrow S_{\mathrm{FAST}}{}^{unit}\;\;descent \end{array}\right.$$

At this point, it is very likely that there will be between one and three unit descent directions in the neighborhood of the current best record. Sensitivities are hence defined as follows (j = 1,2,…NMP):

(22) $$\frac{\partial Ω}{\partial x_{j}}=\mathrm{Min}\left\{\begin{array}{l} \frac{\left[\Omega \left(X_{\mathrm{GR}}{}^{\left(1\right)}\right)-\Omega_{\mathrm{OPT}}\right]}{\left\|S_{\mathrm{BEST}}{}^{unit}\right\|}\frac{\left(x_{\mathrm{GR},j}{}^{\left(1\right)}-x_{\mathrm{OPT},j}\right)}{\left\|S_{\mathrm{BEST}}{}^{unit}\right\|};\frac{\left[\Omega \left(X_{\mathrm{GR}}{}^{\left(2\right)}\right)-\Omega_{\mathrm{OPT}}\right]}{\left\|S_{\text{2ndBEST}}{}^{unit}\right\|}\frac{\left(x_{\mathrm{GR},j}{}^{\left(2\right)}-x_{\mathrm{OPT},j}\right)}{\left\|S_{\text{2ndBEST}}{}^{unit}\right\|};\\ \frac{\left[\Omega \left(X_{\mathrm{GR}}{}^{\left(3\right)}\right)-\Omega_{\mathrm{OPT}}\right]}{\left\|S_{\mathrm{FAST}}{}^{unit}\right\|}\frac{\left(x_{\mathrm{GR},j}{}^{\left(3\right)}-x_{\mathrm{OPT},j}\right)}{\left\|S_{\mathrm{FAST}}{}^{unit}\right\|} \end{array}\right\}$$

Equation (22) shows that the directional derivative along a unit direction is scaled by the direction cosines in order to get sensitivities with respect to optimization variables. The minimum in Equation (22) accounts for the possibility of having non-descent unit directions. In the limit case of three non-descent directions, sensitivity is set equal to the minimum positive value so as to minimize the cost function increment in the neighborhood of the current best record. The approximate gradient evaluation strategy implemented by HFHS allows computational cost of the identification process to be significantly reduced with respect to previously developed HS variants. Once derivatives are computed, Step 1 is completed in the same way as described before.

### 3.2. Step 2: Evaluation of The New Trial Design

The quality of the new harmony **X_TR_** defined in Step 1 is evaluated in this step. In classical HS, if the new trial design **X_TR_** is better than the worst design **X_WORST_** currently stored in the harmony memory, it replaces the worst design in [HM]. The sophisticated generation mechanism developed in this research makes the new trial design have a high probability of improving also the current best record. The following cases may occur: (i) Ω(**X_TR_**) < Ω**_OPT_**; (ii) Ω(**X_TR_**) > Ω**_OPT_**.

If Ω(**X_TR_**) < Ω**_OPT_**, the worst design is removed and the new harmony **X_TR_** is set as the current best record. The former optimum design becomes the second best design stored in the population. The remaining (N_POP_ − 2) designs are analyzed. Let (**X**_NPOP−2_)^r^ be a generic harmony of these (N_POP_ − 2) designs. For each remaining harmony (**X**_NPOP−2_)^r^, the approximate gradient with respect to the current optimum is determined as ΔΩ^r^/ΔS^r^, where ΔΩ^r^ = [Ω((**X**_NPOP−2_)^r^) − Ω**_OPT_**] and ΔS^r^ = ||(**X**_NPOP−2_)^r^ − **X_OPT_**||. Let **X**_NPOP−2_^FAST^ be the harmony corresponding to the largest approximate gradient. Each (**X**_NPOP−2_)^r^ harmony is tentatively updated using Equation (23), with r∈(N_POP_ − 2):

(**X**_NPOP−2_)^r,new^ = (**X**_NPOP−2_)^r^ + η_BEST_ × [**X**_OPT_ − (**X**_NPOP−2_)^r^] + η_2ndBEST_ × [**X**_OPT_ − (**X**_NPOP−2_)^r^] + η_FAST_ × [(**X**_OPT_ − **X**_NPOP−2_^FAST^)] (23)

where η_BEST_, η_2ndBEST_ and η_FAST_ are three random numbers extracted in the (0,1) interval.

If Ω((**X**_NPOP−2_)^r,new^) < Ω((**X**_NPOP−2_)^r^), the new harmony (**X**_NPOP−2_)^r,new^ replaces the old harmony (**X**_NPOP−2_)^r^. Otherwise, the new harmony is discarded and the hold harmony is kept in the population. The population is reordered based on the cost of each harmony. Equation (23) introduces a sort of social behavior that induces harmonies to approach the two best designs stored in the population and to reduce the cost function as fastest as possible.

If Ω(**X_TR_**) > Ω**_OPT_**, the new harmony **X_TR_** is compared with the rest of the population. Let us assume that **X_TR_** ranks p^th^ in the population of N_POP_ designs. The former worst design is removed from the population and the former second worst design becomes the new worst design. Hence, there are (p − 1) better designs than **X_TR_** and (N_POP_ − p) worse designs than **X_TR_**.

The (N_POP_ − p) designs are analyzed similarly to what is done for Ω(**X_TR_**) < Ω**_OPT_**. New harmonies are defined using Equation (24), with r∈(N_POP_−p):

(**X**_NPOP−p_)^r,new^ = (**X**_NPOP−p_)^r^ + η_BEST_·[**X**_OPT_ − (**X**_NPOP−p_)^r^] + η_2ndBEST_·[**X**_OPT_ − (**X**_NPOP−p_)^r^] + η_FAST_·[(**X**_OPT_ − **X**_NPOP−p_^FAST^)] (24)

where η_BEST_, η_2ndBEST_ and η_FAST_ are three random numbers in the (0,1) interval. The new harmony (**X**_NPOP−p_)^r,new^ replaces the old harmony (**X**_NPOP−p_)^r^ if it yields a lower value of error functional. The population is reordered based on the new values of Ω.

This strategy has the following rationale. Whilst **X_TR_** could not replace the optimum, it has a higher quality than other designs of the population. Hence, the other individuals try to imitate its behavior, at least approaching the optimum and improving their positions.

### 3.3. Step 3: Perform 1-D “Local” Annealing Search

If Step 2 could not improve **X**_OPT_, the 1-D “local” annealing search mechanism described in Section 2 is utilized. Variables are perturbed in the neighborhood of **X**_OPT_ based on the magnitude of sensitivities ∂Ω/∂x_j_. Trial designs that yield a positive increment ΔΩ_s_ > 0 with respect to Ω_OPT_ and satisfy the Metropolis’ criterion replace the worst designs stored in the harmony memory [HM].

### 3.4. Step 4: Check for Convergence

As the optimization process proceeds towards the global optimum, population sparsity must decrease. For this reason, the “average” design is defined as **X_aver_** = $\left(\sum_{k=1}^{N_{\mathrm{POP}}}X_{k}\right)/N_{\mathrm{POP}}$. The average value of error functional Ω_aver_ is defined as Ω_aver_ = $\left(\sum_{k=1}^{N_{\mathrm{POP}}}\Omega (X_{k})\right)\;/N_{\mathrm{POP}}$.

The following termination criterion is utilized in this research:

(25) $$\mathrm{Max}\left\{\begin{array}{l} \frac{\mathrm{STD}\left\{\;\left\|X_{1}-X_{\mathrm{aver}}\right\|\;,\left\|X_{2}-X_{\mathrm{aver}}\right\|\;,\ldots ,\left\|X_{\mathrm{NPOP}}-X_{\mathrm{aver}}\right\|\;\right\}}{||X_{\mathrm{aver}}||\;};\\ \frac{\mathrm{STD}\left\{\Omega_{1},\;\Omega_{2},\ldots ,\;\Omega_{{}^{k}},\ldots ,\;\Omega_{\mathrm{NPOP}}\right\}}{\Omega_{aver}} \end{array}\right\}\leq \varepsilon_{\mathrm{CONV}}$$

where the convergence limit ε_CONV_ is set equal to 10^−15^, smaller than the double precision limit used in computing technology. Steps 1 to 4 are repeated until the HFHS algorithm converges to the global optimum.

### 3.5. Step 5: End Optimization Process

The present HFHS algorithm terminates the optimization process and writes the output data in the results file.

## 4. Hybrid Fast Big Bang-Big Crunch

**Figure 4:**
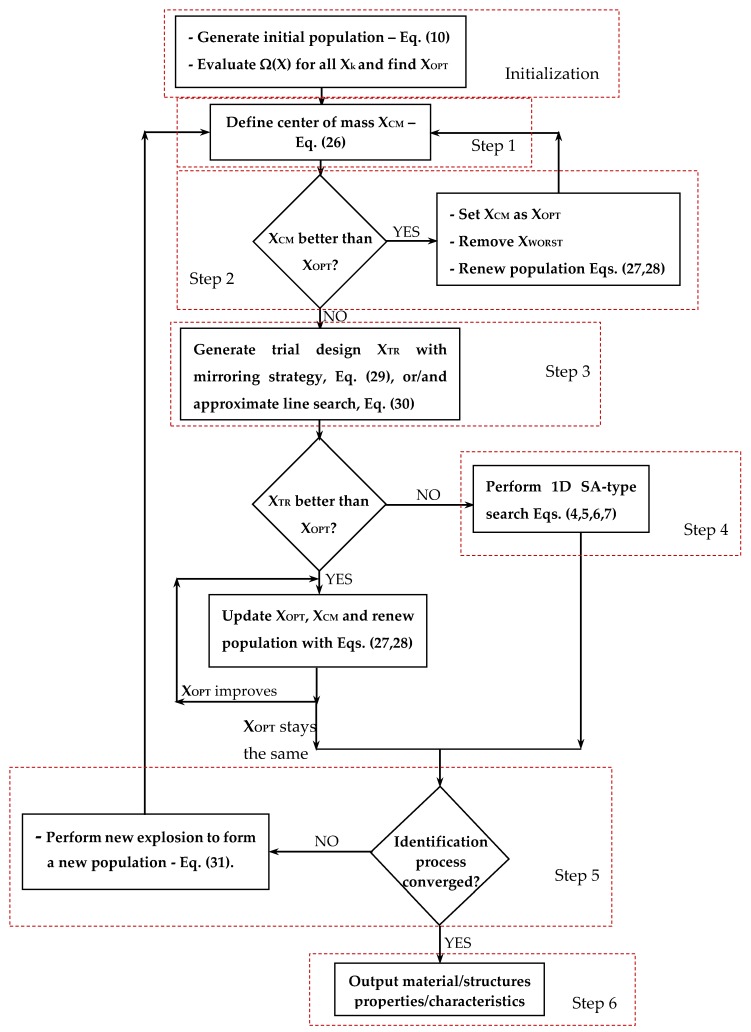
Flow chart of the HFBBBC algorithm developed in this research.

The new HFBBBC algorithm developed in this research is described in detail in this section. The strength points of the present formulation with respect to state-of-the-art BBBC variants can be summarized as follows. First, similar to HFSA and HFHS, computation of sensitivities does not entail new structural analyses. Second, descent directions from which **X_TR_** is generated are more accurately selected. Third, population is dynamically updated so as to simulate an explosion about the center of mass but with all new trial designs lying on potentially descent directions. The hybrid nature of the HFBBBC algorithm comes from the combination of the explosion/contraction process with 1-D local annealing and line search mechanisms. The flow chart of the algorithm is shown in Figure 4.

Like HFHS, the initial population of N_POP_ designs used by HFBBBC is generated with Equation (10). The present algorithm does not require any setting of internal parameters except for the population size N_POP_. Error functional is evaluated for all candidate solutions. The best design **X_OPT_** corresponding to the lowest value of error functional Ω**_OPT_** is determined.

### 4.1. Step 1: Definition of The Center of Mass

The coordinates of the center of mass of the population **X_CM_**(x_CM,1,_x_CM,2,…,_x_CM,NMP_) are defined as:

(26) $$x_{\mathrm{CM},j}=\left(\sum_{k=1}^{N_{\mathrm{POP}}}\frac{x_{j}{}^{k}}{\Omega^{k}}\right)/\left(\sum_{k=1}^{N_{\mathrm{POP}}}\frac{1}{\Omega^{k}}\right)\;\;\;(j\;=\;1,\ldots ,\mathrm{NMP})$$

where x_j,_^k^ is the value of the j^th^ optimization variable stored in the k^th^ trial design, Ω^k^ is error functional value for the k^th^ trial design. Penalty functions can be used to sort designs. The weighting coefficients 1/Ω^k^ make position of center mass be more sensitive to the best designs stored in the population.

### 4.2. Step 2: Evaluation of The Center of Mass and Progressive Update of X_CM_ As Current Best Record

Error functional is evaluated at **X_CM_**. As mentioned above, BBBC formulations usually converge to the optimum design by updating the position of **X_CM_**. However, there is no guarantee that the new **X_CM_** may be the center of a better population. Since **X_CM_** represents a weighted average of candidate designs, its quality will be somewhere in between the worst and best individuals included in the population. The present HFBBBC algorithm considers two cases: (i) **X_CM_** is better than **X_OPT_**; (ii) **X_CM_** is worse than **X_OPT_**.

In [164,165,167], **X_CM_** was reset as **X_OPT_** if case (i) occurred. The worst design included in the population was replaced by **X_CM_** and a new center of mass was defined. The same was done until case (ii) occurred. That approach allows one to avoid performing a new explosion about each new center of mass, thus saving N_POP_ structural analyses with respect to classical BBBC. However, it was replaced only one design at a time while classical BBBC renews the whole population each time **X_CM_** is updated. In order to overcome this limitation without increasing computational cost, the following strategy has been implemented in this study.

If **X_CM_** is better than **X_OPT_**, it is reset as **X_OPT_**. The former best record becomes the second best design. The worst design is removed from the population. Any direction defined as (**X_OPT_** − **X_k_**) is a descent direction with respect to **X_k_** because Ω(**X_k_**) > Ω(**X_OPT_**): the design improves as we move away from **X_k_**. However, (**X_OPT_** − **X_k_**) is also opposite to (**X_k_** − **X_OPT_**), which is a non-descent direction with respect to **X_OPT_**. If cost functional gradient changes smoothly, a direction which was descent for **X_k_** may remain descent also for **X_OPT_**. In view of this, HFBBBC tentatively updates designs as:

**X_k_**^tentative^ = **X_k_** + (1 + ξ_k_)(**X_OPT_** − **X_k_**)    (k = 1,…,N_POP_ − 1) (27)

where ξ_k_ is a random number in the interval (−1,1). If ξ_k_∈(−1,0), **X_k_**^tentative^ lies between **X_k_** and **X_OPT_**; if ξ_k_∈(0,1), **X_k_**^tentative^ lies beyond **X_OPT_**.

Since the **X_k_**^tentative^ designs are potentially better than the **X_k_** designs as they have been defined by moving towards the current best record or trying to improve **X_OPT_** itself, a population including the (N_POP_ − 1) designs **X_k_**^tentative^ and the current best record **X_OPT_** should be of higher quality than the current population. Consequently, the new center of mass **X_CM_**^tentative^ should be better than the former center of mass **X_CM_** defined for the original population and further improve **X_OPT_**.

In order to reduce computational cost, approximate values of error functional Ω_APP_(**X_k_**^tentative^) are determined as:

(28) $$\left\{ \begin{array}{ll} \Omega \left(X_{k}{}^{tentative}\right)=\Omega \left(X_{k}\right)\;\frac{\left||X_{k}{}^{tentative}-X_{k}|\right|}{\left||X_{\mathrm{OPT}}-X_{k}|\right|} & if\;\xi_{k}\in (-1,0)\\ \Omega \left(X_{k}{}^{tentative}\right)=\Omega \left(X_{k}\right)\;\frac{\left||X_{k}{}^{tentative}-X_{\mathrm{OPT}}|\right|}{\left||X_{\mathrm{OPT}}-X_{k}|\right|} & if\;\xi_{k}\in (0,1) \end{array}\right.\;,\;(k\;=\;1,2,\ldots ,N_{\mathrm{POP}}\;-\;1)$$

The approximate position of the center of mass **X_CM_**^tentative^ is determined with Equation (26) using the **X_k_**^tentative^ vectors and the approximate values of error functional Ω_APP_(**X_k_**^tentative^). The real value of error functional is evaluated at **X_CM_**^tentative^. If **X_CM_**^tentative^ is better than **X_OPT_**, it is reset as **X_OPT_**. The **X_k_**^tentative^ designs replace the original designs **X_k_** and a new loop is performed using Equations (27) and (28). If **X_CM_**^tentative^ does not improve any more the current best record **X_OPT_**, a new center of mass **(X_CM_^tentative^)’** is defined by changing only the weights of the designs that lie between **X_k_** and **X_OPT_**: that is, Equation (27) is used only for ξ_k_∈(−1,0). This is done because the **X_k_**^tentative^ designs lying beyond **X_OPT_** could violate side constraints because of the very large perturbations given to variables.

If **(X_CM_^tentative^)’** improves the current best record, it is reset as **X_OPT_**. The **X_k_**^tentative^ designs generated for ξ_k_∈(−1,0) replace the corresponding **X_k_** designs. A new loop is performed using Equations (27) and (28). This process is repeated until a new center of mass improves the current best record.

If both points **X_CM_**^tentative^ and **(X_CM_^tentative^)’** do not improve **X_OPT_**, the **X_k_**^tentative^ designs are moved back to the corresponding **X_k_** designs and Step 3 is executed.

Similar to classical BBBC, population is renewed each time the position of the center of mass is updated. However, the present algorithm requires only one or two structural analyses to evaluate **X_CM_**^tentative^ and **(X_CM_^tentative^)’** vs. between the rather broad range of 0.1N_POP_ to N_POP_ analyses (often sensitive to the optimization problem at hand) required by state-of-the-art BBBC algorithms (see for example [169]).

### 4.3. Step 3: Evaluation of The Center of Mass and Formation of new Trial Designs Different from X_CM_

The case Ω(**X_CM_**) > Ω(**X_OPT_**) (i.e., **X_CM_** is worse than **X_OPT_**) is the most likely to occur because the center of mass averages the properties of the N_POP_ designs included in the population and hence it should rank between **X_WORST_** and **X_OPT_**. The present algorithm utilizes a computationally inexpensive approach. A new trial design **X_TR_^mirr^** is defined with the mirroring strategy. That is:

**X_TR_^mirr^** = (1 + η_MIRR_)⋅**X_OPT_**−η_MIRR_ × **X_CM_** (29)

where η_MIRR_ is a random number in the interval (0,1). The mirroring strategy attempts to turn the non-descent direction (**X_CM_** − **X_OPT_**) into the descent direction (**X_TR_^mirr^** − **X_OPT_**). Using a random number smaller than one limits the search in the neighborhood of the current best record.

If Ω (**X_TR_^mirr^**) < Ω (**X_OPT_**), this trial design replaces the current best record which becomes the second best design of the population. The worst design is removed from the population. The optimization process is continued with Step 2 to generate (N_POP_ − 1) **X_k_**^tentative^ designs, renew population and update position of **X_CM_**; convergence is checked in Step 5.

If Ω(**X_TR_^mirr^**) > Ω(**X_OPT_**), the mirroring strategy (29) is judged not effective and a new trial design must be generated by combining a set of descent directions. Since Ω(**X_CM_**) > Ω(**X_OPT_**), the (**X_CM_** − **X_OPT_**) vector is a non-descent direction with respect to the current best record. However, the opposite direction **S_OPT_**_–**CM**_ = −(**X_CM_** − **X_OPT_**) ≡ (**X_OPT_** − **X_CM_**) may be a descent direction, especially if the gradient of cost function is smooth. Similar to the HFHS algorithm described in Section 3, the approximate gradient of Ω(**X**) is determined also for HFBBBC. All (**X_OPT_** − **X_k_**) vectors are opposite to directions (**X_k_** − **X_OPT_**) that were non-descent with respect to **X_OPT_**. For each design **X_k_** the approximate gradient ∇Ω_k_^appr^ = [Ω(**X_k_**) − Ω(**X_OPT_**)]/||**X_k_** − **X_OPT_**|| is calculated. The **S_BEST_** = (**X_OPT_** − **X_WORST_**) direction corresponding to the largest variation of cost function between two candidate designs, the **S_FAST_** = (**X_OPT_** − **X_FAST_**) direction corresponding to the largest ∇Ω_k_^appr^, and the **S_2ndBEST_** = (**X_OPT_** − **X_2ndBEST_**) direction corresponding to the second best design are considered. A new trial design **X_TR_** is defined as:

**X**_TR_ = **X_OPT_** + η_OPT−CM_**S_OPT_**_–**CM**_ + η_BEST_**S_BEST_** + η_2ndBEST_**S_2ndBEST_** + η_FAST_**S_FAST_** (30)

where η_OPT–CM_, η_BEST_, η_2ndBEST_ and η_FAST_ are four random numbers generated in the (0,1) interval.

**Figure 5:**
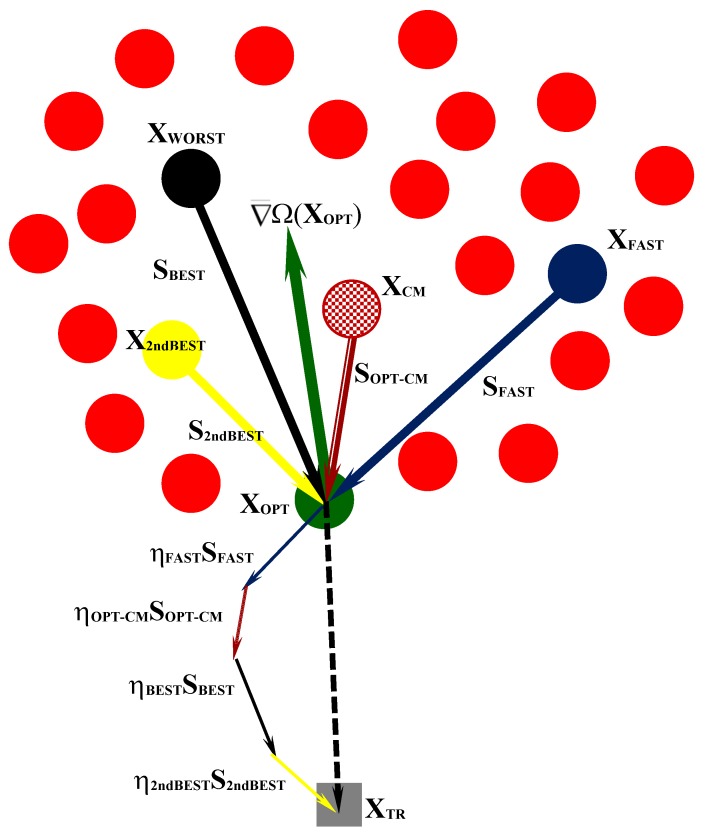
Determination of trial points for hybrid HFBBBC when Ω(**X_CM_**) > Ω(**X_OPT_**). The red dots represent designs stored in the population.

The generation of a new trial solution **X_TR_** with Equation (30) is illustrated in Figure 5 for an inverse problem with two variables. If the error functional gradient is smooth enough, a descent direction **S** will satisfy the condition **S^T^**$\bar{\nabla }$Ω(**X_OPT_**) < 0, as it appears to be for **S_OPT_**_–**CM**_, **S_BEST_**, **S_FAST_** and **S_2ndBEST_** directions in the figure. By summing up the steps taken on **S_OPT_**_–**CM**_, **S_BEST_**, **S_FAST_** and **S_2ndBEST_**, a trial design **X_TR_** lying on a descent direction can be obtained.

The present HFBBBC algorithm directly perturbs variables with respect to the current best record and hence generates higher quality designs. The effect of the average properties of the population described by **X_CM_** is now taken into account by considering the **S_OPT_**_–**CM**_ direction.

The quality of **X_TR_** is evaluated as usual. If Ω(**X_TR_**) < Ω(**X_OPT_**), the trial design replaces the current best record and the worst design is removed from the population. Step 2 is performed to eventually renew population and update position of **X_CM_**; convergence check is performed in Step 5. Otherwise, the 1-D “local” annealing search of Step 4 is executed.

### 4.4. Step 4: Perform 1-D “Local” Annealing Search

HFBBBC utilizes the same probabilistic search mechanism implemented in HFSA and HFHS. However, the position of the center of mass is updated each time a trial design improves the current best record. The new center of mass and its mirror point with respect to the current best record also are evaluated to check for further improvements in design or to define additional points satisfying the Metropolis criterion.

### 4.5. Step 5: Check for Convergence and Perform A New Explosion If Necessary

HFBBBC checks if the best design of the population has been improved in the current optimization cycle. Convergence check is performed after operations entailed by Steps 3 and 4. Let be (**X_OPT_**)^init^ and (**X_OPT_**)^final^ the best designs at the beginning and at the end of the current optimization cycle. If (**X_OPT_**)^final^ is better than (**X_OPT_**)^init^, HFBBBC checks for convergence using the same criterion, Equation (25), adopted for HFHS. If convergence is reached, Step 6 is executed. Otherwise, Steps 1 to 4 are repeated until HFBBBC converges to the global optimum.

If (**X_OPT_**)^init^ is equal to (**X_OPT_**)^final^, the current optimization cycle did not improve design in spite of the numerous improvement routines available in HFBBBC. For this reason, a new explosion is performed about **X_OPT_** trying to generate a higher quality population. The following equation is utilized:

(31) $$x_{j}^{k}=x_{\mathrm{OPT},j}-\rho_{j}{}^{k}\;(x_{\mathrm{OPT},j}-x_{\mathrm{CM},j})\;\;\;(k\;=\;1,2,\ldots ,N_{\mathrm{POP}};\;j\;=\;1,2,\ldots ,\mathrm{NMP})$$

where ρj^k^ is a random number in the interval (0,2) to generate the j^th^ variable of the k^th^ design. The interval (0,2) is large enough to avoid stagnation near the current best record. If $x_{j}^{k}<\;x_{j}^{L}$ or $x_{j}^{k}>\;x_{j}^{U}$, $x_{j}^{k}$ is reset to $x_{j}^{k}=(\;x_{j}^{L}+x_{\mathrm{OPT},j})/2$ or $x_{j}^{k}=(\;x_{\mathrm{OPT},j}+x_{j}^{U})/2$, respectively. The new population is generated by perturbing the current best record **X_OPT_** along the direction −(**X_OPT_** − **X_CM_**), opposite to the non-descent direction (**X_CM_** − **X_OPT_**). The rationale of Equation (31) is to search for descent directions with respect to **X_OPT_** by decomposing a potentially descent direction in its components.

The new designs are compared with the previous population and only the best N_POP_ designs are retained in the new population. This elitist strategy allows to keep the **X_OPT_** design in the population passed into the next optimization iteration should all of the new N_POP_ designs generated with Equation (31) be worse than **X_OPT_**. The optimization process is reprised from Step 1.

### 4.6. Step 6: End Optimization Process

The HFBBBC algorithm terminates the optimization process and writes the output data in the results file.

## 5. Test Problems and Results

The HFSA, HFHS and HFBBBC algorithms developed in this study for mechanical identification problems were tested on two composite structures and a hyperelastic biological membrane. They were compared with other SA/HS/BBBC variants (e.g., [77,81,83,84] and their successive enhancements [162,163,164,165,166]) including gradient information in the optimization search, as well as with adaptive harmony search [170,171], big bang-big crunch with upper bound strategy (BBBC-UBS) [172], JAYA [35], MATLAB Sequential Quadratic Programming (MATLAB-SQP) [173] and ANSYS built-in optimization routines [174]. The ANSYS built-in optimization routines (e.g., gradient-based, zero order and response surface approximation) were run in cascade or alternated in order to maximize their efficiency.

The abovementioned comparison should be considered very indicative for the following reasons:

- Adaptive HS [170,171] and BBBC-UBS [172] represent state-of-the-art formulations of harmony search and big bang-big crunch, which have been successfully utilized in many optimization problems. In particular, the adaptive HS algorithm adaptively changes internal parameters without any intervention by the user: this approach is very similar to what is done by HFHS. The BBBC-UBS algorithm [172] immediately discharges trial designs that certainly would not improve the current best design included in the population, thus saving computational cost; this elitist strategy is somehow consistent with the rationale followed by HFSA, HFHS and HFBBBC that always try to generate trial designs lying on descent directions.
- JAYA [35] is one of the most recently developed metaheuristic algorithms that has soon emerged as a very powerful method and gathered great consideration from optimization experts. The basic idea of JAYA is very simple yet very effective: search process tries to move toward the best design and avoid the worst design of the population. Besides this, JAYA is very easy to implement and does not have internal parameters to be tuned. The basic formulation of JAYA was successfully used in the damage detection problems solved in [153,154]. In [175,176], JAYA’s computational efficiency was improved by adding an elitist strategy, which is conceptually similar to that used by BBBC-UBS. However, such a strategy may become computationally ineffective for inverse problems as it entails a new finite element analysis each time a design of the population is updated. Nevertheless, it is interesting to compare HFSA, HFHS and HFBBBC with JAYA also.
- SQP is universally reputed by optimization experts the best gradient-based method available in the literature. The method is globally convergent, does not require setting of move limits and does not suffer from premature convergence. The successful use of MATLAB-SQP in highly nonlinear inverse problems taken from very different fields (e.g., optical super-resolution with evanescent illumination, visco-hyperelasticity of cell membranes, damage detection etc.) is well documented in the literature (see, for example, [11,12,14,177,178,179]).

The structural analyses entailed by the optimization process to evaluate the error functional Ω were performed with the commercial finite element program ANSYS^®^ [174]. Each metaheuristic search engine and MATLAB-SQP were properly interfaced with the finite element solver. Since the gradient of error functional is not explicitly available and evaluating Ω entails structural analyses, the present algorithms computed approximate gradients as described in Section 2, Section 3 and Section 4. Partial derivatives ∂Ω/∂X_i_ (i = 1,…,NMP) and $\partial \delta_{\mathrm{FEM}}{}^{j}/\partial X_{i}$ required by previously developed SA variants [77,81,83,84] were instead evaluated with centered finite differences (δX_i_ = X_OPT,i_/10,000). Consequently, weighting coefficients μ_i_ = (∂Ω/∂X_i_)/||$\bar{\nabla }$Ω(**X_OPT_**)|| were determined as:

$$\sum_{j=1}^{N_{\mathrm{CNT}}}\left[{\left|\left(\delta_{\mathrm{FEM}}{}^{j}-\bar{\delta^{j}}\right)/\bar{\delta^{j}}\right|}^{2}\cdot \frac{\partial \delta^{j}}{\partial X_{i}}\right]/\sqrt{N_{\mathrm{CNT}}\cdot \Omega (X_{\mathrm{OPT},1},X_{\mathrm{OPT},2},\ldots ,X_{\mathrm{OPT},i},\ldots ,X_{\mathrm{OPT},\mathrm{NMP}}).}$$

SQP-MATLAB computed the $\bar{\nabla }$Ω(**X_OPT_**) gradient with forward finite differences and progressively updated the [B] matrix involved in the (**S**^T^[B]**S**)/2 term of the quadratic approximation of the error functional Ω. The search direction **S** represents the solution of the approximate sub-problem built in each iteration. The [B] matrix is initially set equal to the unit matrix and finally converges to the Hessian matrix of the error functional Ω.

Before running optimizations with the new algorithms HFSA, HFHS and HFBBBC, we tried to simplify the previously developed SA/HS/BBBC formulations [77,81,83,84,162,163,164,165,166] adapting them to inverse problems. The goal was to drastically reduce the number of structural analyses required by the identification process. For example, in the case of SA variants used in [77,81,83,84], the global annealing search equation involving sensitivities ∂Ω/∂X_i_ is replaced by:

X_i_^TR^ = X_OPT,i_ + (X_i_^U^ − X_i_^L^) ρ_I_ × Ω_OPT,*l*−1_/Ω_OPT,*l*_    (i = 1,…,NMP) (32)

The new trial design **X_TR_** thus obtained is evaluated with respect to the current best record **X_OPT_**. If Ω(**X_TR_**) < Ω(**X_OPT_**), **X_TR_** is reset as the current best record and a new trial point is defined with Eq. (32). Conversely, if Ω(**X_TR_**) > Ω(**X_OPT_**), a new trial point **X_TR_^new^** = **2X_OPT_** − **X_TR_** is defined via mirroring strategy and evaluated with respect to **X_OPT_**.

If Ω(**X_TR_^new^**) < Ω(**X_OPT_**), **X_TR_^new^** is reset as **X_OPT_** and a new trial design is generated with Equation (32). Conversely, if it still holds Ω(**X_TR_^new^**) > Ω(**X_OPT_**), the error functional Ω(**X**) is approximated by a 4th order polynomial Ω_APP_(**X**) passing through the five trial points **X_TR_**, **X_INT_^’^**, **X_OPT_**, **X_INT_^’’^** and **X_TR_^new^** where **X_INT_^’^** is a randomly generated trial point between **X_TR_** and **X_OPT_** while **X_INT_^’’^** is another randomly generated trial point between **X_OPT_** and **X_TR_^new^**. If there is a point X_OPT_* minimizing the approximate error functional in the segment limited by **X_TR_** and **X_TR_^new^**, a new exact structural analysis is performed to compute Ω(X_OPT_*). If Ω(X_OPT_*) > Ω(**X_OPT_**), trial designs **X_TR_**, **X_INT_^’^**, X_OPT_*, **X_INT_^’’^** and **X_TR_^new^** are accepted or rejected based on Metropolis’ criterion. Finally, the trial design with the smallest value of error functional is reset as **X_OPT_**. If Ω_APP_(**X**) does not have any minima, the 1-D local annealing search is performed until the current best record is updated.

The above described SA strategy—denoted as SA-NGR in the rest of this article—does not require any exact gradient evaluation but it includes a rather simple line search strategy, which does not ensure trial designs to be lying on descent directions. The (X_i_^U^ − X_i_^L^) step used in Equation (32) may result in larger perturbations and hence less optimization cycles. However, the total number of structural analyses required in the identification process does not change substantially with respect to the SA variants including gradient evaluations [77,81,83,84].

In the case of HS algorithm, any trial design **X_TR_** is defined as:

**X**_TR_ = **X**_OPT_ + ρ_FAST_**S**_FAST_ + ρ_BEST_**S**_BEST_ (33)

where: **S_FAST_** and **S_BEST_**, respectively, are the steepest descent and the best directions moving from population designs towards the current best record **X_OPT_** (the same nomenclature used for the derivation of Equation (22) applies also in this case); ρ_FAST_ and ρ_BEST_ are two random numbers in the interval (0,1), respectively, generated for **S_FAST_** and **S_BEST_**. Unlike HS variants [164,166,167], Equation (33) directly utilizes approximate line search to define descent directions.

If the new trial design **X_TR_** is better than the worst design **X_WORST_** included in the harmony memory matrix [HM], it replaces it and the updated population is re-ordered to determine the new current best record. Conversely, if Ω(**X_TR_**) > Ω(**X_WORST_**), a new trial point **X_TR_^new^** = **2·X_OPT_** − **X_TR_** is defined via mirroring strategy. If it holds again Ω(**X_TR_^new^**) > Ω (**X_WORST_**) (this may be due to nonlinearity and non-convexity of the inverse problem), the 4th order polynomial approximation of Ω described above is performed to find a point of minimum X* yielding Ω(X*) < Ω(**X_WORST_**). Should this search be unsuccessful, mirroring strategy and approximate line search are repeated until a trial design better than the worst design stored in the harmony memory is found.

Similar to SA-NGR, this simplified HS formulation—denoted as HS-NGR in the rest of this article—does not evaluate the gradient of error functional. However, it considers only two potentially descent directions (yet defined from approximate line search) and hence it is forced to repeatedly perform mirroring of trial designs, polynomial approximation of error functional and 1-D annealing search. While the classical HS strategy of replacing only the worst design is adopted also by HS-NGR without following any elitist criterion, the main architecture of HS based on the use of HMCR and bandwidth parameters is not retained. In fact, HS-NGR always uses the same Equation (33) to generate new trial designs regardless of the fact that the current trend of variation of the error functional would suggest performing exploitation rather than exploration. Consequently, HS-NGR may be unsuccessful in global search and it attempts to correct this problem by carrying out a local search, which usually entails many structural analyses. Furthermore, replacing only the worst design results in an extra number of FE analyses. This counterbalances the reduction in the number of analyses achieved by not computing gradients via finite differences.

In the case of the BBBC algorithm, any trial design **X_TR_** is always defined as:

**X**_TR_ = **X**_CM_ + ρ_FAST,CM_ × **S**_FAST,CM_ + ρ_BEST,CM_ × **S**_BEST,CM_ (34)

where: **S_FAST,CM_** and **S_BEST,CM_**, respectively, are the steepest descent and the best directions moving towards the center of mass of the population (definitions are the same as for Equation (22) but **X_OPT_** is replaced by **X_CM_**); ρ_FAST_ and ρ_BEST_ are two random numbers in the interval (0,1), respectively, generated for **S_FAST,CM_** and **S_BEST,CM_**. Unlike BBBC variants [165,166] and similar to Equation (33) used for HS-NGR, Equation (34) directly utilizes approximate line search to define descent directions.

The new trial design **X_TR_** is evaluated in the same way as in the SA-NGR algorithm and a new explosion in the neighborhood of the center of mass is performed only if the current **X_OPT_** could not be improved.

Since the above described simplified BBBC variant is a gradient free algorithm, it will be denoted as BBBC-NGR in the rest of the article. At first glance, BBBC-NGR has to deal with two critical aspects: (i) solution is perturbed with respect to the center of mass of the population rather than with respect to the current best record; (ii) a limited number of potentially descent directions are considered in the formation of a new trial solution. Consequently, the reduction of computational cost granted by the smaller number of explosions may by counterbalanced by the additional structural analyses performed in the attempt of improving current best record with mirroring strategy and 4th order approximation of error functional Ω.

### 5.1. Mathematical Optimization Benchmark: Random Minimum Square Problem

The inverse problem (1) basically is a least square problem. A randomized version of the problem can be stated in the general form for NMP design variables as:

(35) $$\{\begin{array}{ll} \mathrm{Min}\;\Omega & =\overset{\mathrm{NMP}}{\underset{i=1}{\sum }}\begin{array}{c} {\left(x_{i}-\eta_{i}\right)}^{2} \end{array}\\ & -1\leq x_{i}\leq 1 \end{array}$$

where η_i_ (i = 1,…,NMP) are random numbers generated in the (−1,1) interval. The cost function of this problem is unimodal and has a global minimum located at **X_TRG_**(η_1_,η_2_,…,η_NMP_) and leading to Ω_MIN_ = 0. The random numbers η_i_ in Equation (35) introduce noise in the least square optimization process similar to the noise that may be caused by optical measurements. Here, the target vector **X_TRG_**(η_1_,η_2_,…,η_NMP_) was selected by averaging five randomly generated vectors.

In order to carry out a preliminary comparison between the present algorithms and other optimizers, the problem (35) was solved with NMP = 100 or NMP = 500 using HFSA, HFHS, HFBBBC, adaptive HS [170,171], BBBC-UBS [172], JAYA [35,175,176] and SQP-MATLAB. Using NMP = 500 variables allowed to simulate the use of a fairly large number of control points at which the optically measured displacements are compared with finite element results.

The population size for HFHS, HFBBBC and JAYA was set as 20, 200, 500 and 1000 in order to analyze sensitivity of convergence behavior to N_POP_. Because of the random nature of metaheuristic search engines, 20 independent optimization runs were carried out for each setting of N_POP_ and NMP. HFSA and SQP-MATLAB runs were started from the best point and center of mass of each initial population defined for HFHS, HFBBBC and JAYA. These points were very far from the target solution: in fact, initial values of Ω ranged between 26.33 and 34.15 with an average percent error on variables ranging between 541.4% and 1392.7%.

The present algorithms found very competitive designs with SQP-MATLAB but required up to three function evaluations to complete the optimization process: on average, 3196 (HFSA), 3562 (HFHS) and 3813 (HFBBBC) vs. 1650 (SQP-MATLAB). However, the average optimized cost and standard deviation on optimized cost were significantly smaller for the present algorithms, which converged to more precise solutions than SQP-MATLAB: in particular, (3.798 ± 1.149) × 10^−12^, (2.899 ± 2.623) × 10^−12^ and (7.816 ± 4.241) × 10^−13^, respectively, for HFSA, HFHS and HFBBBC vs. (1.044 ± 0.948) × 10^−10^ obtained by SQP-MATLAB. The higher precision of HFSA, HFHS and HFBBBC is confirmed by the larger deviation of optimized designs from the target solution **X_TRG_** seen in the case of SQP-MATLAB. It should be noted that, since the target optimum **X_TRG_** contains some very small values (for example, of the order of 7 × 10^−4^), even a small difference between some component of **X_OPT_** and **X_TRG_** may make average deviation increase by a large extent.

While convergence behavior of the present SA/HS/BBBC variants was rather insensitive to population size, the efficiency of the gradient-based optimizer decreased for increasing population size due to the larger sparsity of design variable values. Adaptive HS variants [170,171] were outperformed by HFSA, HFHS and HFBBBC as they found some intermediate designs with an average cost of 0.174 after 10000 function evaluations. BBBC-UBS [172] was much more efficient than adaptive HS and its convergence speed improved with population size. However, cost function evaluated after 10000 analyses for BBBC-UBS is still 1.467 × 10^−9^, three orders of magnitude higher than for the present HS/BBBC/SA algorithms. JAYA [35,175,176] was slightly more efficient than BBBC-UBS and arrived at the cost function value of 1.297 × 10^−9^ after 9850 analyses. However, its computational speed significantly decreased with population size.

Results gathered in this preliminary test confirmed the ability of the present algorithms to solve least square type problems including random noise. This conclusion will be proven true in the next sections also for the three identification problems solved in this study.

### 5.2. Woven Composite Laminate

**Figure 6:**
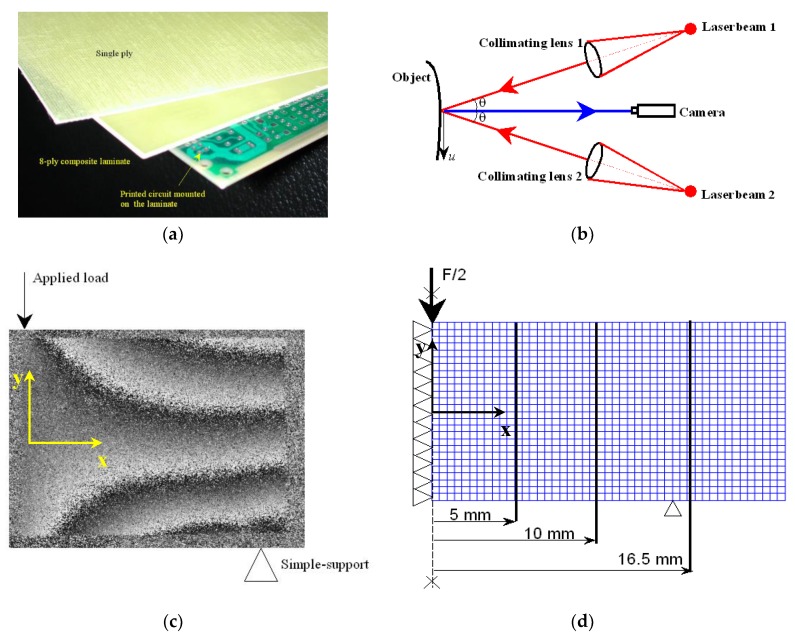
(**a**) Woven composite laminate to be characterized; (**b**) Schematic of ESPI setup sensitive to *u*-displacements; (**c**) Phase pattern of ESPI fringes; (**d**) Finite element model simulating the experiment (control paths are also indicated).

The first inverse problem solved in this study regards the mechanical characterization of an 8-ply woven-reinforced fiberglass-epoxy composite laminate used as substrate for printed circuit boards (see Figure 6a). The error functional Ω to be minimized depends on four unknown elastic constants, E_x_, E_y_, G_xy_ and ν_xy_. The target values of material properties were provided by the industrial partner involved in the project: E_x_ = 25000 MPa, E_y_ = 22000 MPa, G_xy_ = 5000 MPa and ν_xy_ = 0.280.

The optimization process entailed by this identification problem attempts to match the in-plane displacements *u* generated by a vertical load of 140 N that produces 3-point bending. A 46 mm long, 13 mm tall and 1.2 mm thick specimen was cut from the laminate and submitted to 3-point bending. Target displacements *u* included in the error functional Ω were measured with Phase Shifting Electronic Speckle Pattern Interferometry (PS-ESPI) [5,6,7]. The double-illumination interferometer used in the speckle measurements is schematized in Figure 6b; the symmetric illumination beams make the setup be sensitive to *u*-displacements. Illumination is realized with a 35 mW He-Ne laser (λ = 632.8 nm). The angle of illumination θ is 20°. Hence, sensitivity of optical set up is λ/2sinθ = 925.1 nm. Fringe patterns were processed following guidelines illustrated in [180]. More details on the ESPI measurements carried out for this identification problem can be found in [76,77].

The ESPI phase pattern containing displacement information is shown in Figure 6c while Figure 6d shows the finite element model including control paths parallel to the Y-axis of symmetry of the specimen. The specimen was modelled in ANSYS with 4-nodes plane elements under the assumption of plane stress. Element size was selected so as to have mesh independent solutions and nodes located in correspondence of the control points defined on the recorded image. The error functional Ω was built by comparing FE results and experimental data at 78 control points. The following bounds were taken for material parameters in the optimization process: 3000 ≤ E_x_ ≤ 50000 MPa, 2000 ≤ E_y_ ≤ 50000 MPa, 1000 ≤ G_xy_ ≤ 50000 MPa and 0.01 ≤ ν_xy_ ≤ 0.45. These bounds are large enough not to have any effect on the results of the identification problem.

The population size of all HS and BBBC variants considered in this study was set equal to 10, hence 2.5 times as large as the number of unknown material parameters. The same was done for JAYA. Values of Ω corresponding to the best design, worst design and center of mass of the initial population are 0.180, 0.862 and 0.365, respectively. The corresponding average (maximum) deviations from target properties are 33.8% (56.1%), 37.4% (61.1%) and 41% (53%), respectively. HFSA, ANSYS and MATLAB-SQP optimizations were started from each of the three points mentioned above. Thirty independent optimization runs were carried out starting from different initial populations (yet keeping N_POP_ = 10) to statistically evaluate algorithms’ performance.

The results of the identification process are summarized in Table 1. The “SA-Grad” notation refers to the ISA algorithm developed in [77], which combined global and local annealing search strategies based on finite difference evaluation of $\bar{\nabla }$Ω(**X_OPT_**), and was successfully applied to this test case. All HS/BBBC/SA variants determined material properties with a great deal of accuracy. In fact, the largest error, made on the Poisson’s ratio, never exceeded 0.941%. The optimized solutions of HFSA, HFHS and HFBBBC correspond to the lowest errors on material properties. SA-Gradient [77] also was very accurate but required up to 85% more FE analyses than the present algorithms.

The maximum residual error on displacements was always lower than 3%, localized near on the closest control path to the applied load. This happened because the *u*-displacement field is symmetric about the Y-axis (i.e., loading direction) and hence *u*-displacements approach to zero near this axis. The average error on displacements evaluated for the identified material properties was about 0.6% for all algorithms.

Table 1 shows that HFHS and HFBBBC were faster than HFSA as they required, respectively, 210 and 222 structural analyses to complete the optimization process vs. 257 analyses required by HFSA. The proposed algorithms were between 15% and 23% faster than the simplified algorithms SA/HS/BBBC-NGR and the number of explosions and 1-D local annealing searches were substantially reduced by the present formulations. The very small number of optimization variables (only four unknown parameters) defined for this inverse problem somehow limited the ability of HFHS and HFBBBC of building a large number of descent directions.

Remarkably, statistical dispersion on identified material properties, residual error on displacements and required number of finite element analyses evaluated over the thirty independent runs was less than 0.11% thus proving the robustness of the proposed algorithms.

For the sake of brevity, Table 1 does not report the results obtained by AHS [170,171], BBBC-UBS [172], JAYA [35,175,176], MATLAB-SQP [173] and ANSYS [174]. The gradient-based optimizer of ANSYS converged after 48 iterations and about 200 structural analyses to a solution (E_x_ = 24898 MPa; E_y_ = 22306 MPa; G_xy_ = 5225 MPa; ν_xy_ = 0.223) with about 20.3% error on Poisson’s ratio. MATLAB-SQP was more accurate than ANSYS (E_x_ = 25006 MPa; E_y_ = 22026 MPa; G_xy_ = 4971 MPa; ν_xy_ = 0.288) but yet its solution has a 2.8% error on Poisson’s ratio after 45 iterations and about 215 structural analyses. Adaptive HS [170,171] was the slowest algorithm overall: in fact, average error on material properties for the solution E_x_ = 24767 MPa; E_y_ = 21777 MPa; G_xy_ = 5190 MPa; ν_xy_ = 0.279 was still higher than 1.5% after about 400 structural analyses. BBBC-UBS [172] found the solution E_x_ = 25045 MPa; E_y_ = 21991 MPa; G_xy_ = 4889 MPa; ν_xy_ = 0.277 after about 350 structural analyses: this solution is critical with respect to Poisson’s ratio for which there is a 2.2% error. Finally, JAYA [35,175,176] obtained the properties E_x_ = 24991 MPa; E_y_ = 21979 MPa, G_xy_ = 5044 MPa; ν_xy_ = 0.257 after 25 iterations and 250 structural analyses; although elastic moduli were identified very precisely, the Poisson’s ratio error increased to 9.2%.

The above listed data confirm the superiority of the proposed SA/HS/BBBC formulations over metaheuristic algorithms that do not use line search to generate trial solutions belonging to descent directions. The elitist strategies of BBBC-UBS and JAYA are more heuristic and do not form descent directions in a direct way unlike HFHS and HFBBBC.

**Figure 7:**
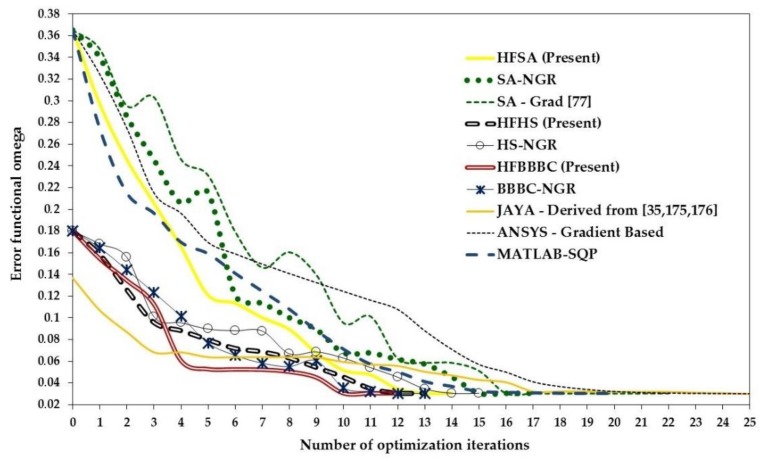
Comparison of convergence behavior of different optimizers in the woven composite laminate identification problem.

The convergence curves obtained for the best optimization runs of HS/BBBC/SA variants, JAYA and gradient-based optimizers are compared in Figure 7. HFBBBC was the fastest algorithm to significantly reduce the error functional but it then had a fairly long step with little improvements in Ω value. This allowed HFSA and HFHS to have an average convergence rate similar to HFBBBC. The small number of design variables made it difficult for HFSA to recover the initial gap in cost function (i.e., 0.180 vs. 0.365). HS-NGR, BBBC-NGR, SA-NGR and SA-Grad showed oscillatory behavior because they formed trial designs considering only one or two potentially descent directions at a time. JAYA’s best run started from a population including better designs than the other algorithms but the search process of this method clearly suffered from the lack of a direct generation of descent directions. In fact, JAYA achieved the last 30% of its total reduction in error functional with respect to the best initial value of Ω = 0.136 over 22 iterations out of a total number of 25 iterations.

MATLAB-SQP was faster than SA-NGR and SA-Grad, comparable in convergence rate with HFSA and HS-NGR for some iterations but definitely slower than HFHS and HFBBBC. Furthermore, its convergence curve became very similar to that of JAYA after 11 iterations. The extra iterations required by JAYA, ANSYS and MATLAB-SQP for completing optimization process were due to their difficulty in converging to the correct value of Poisson’s ratio.

### 5.3. Axially Compressed Composite Panel for Aeronautical Use

The goal of the second inverse problem solved in this study was to identify mechanical properties and ply orientations of a IM7/977-2 graphite-epoxy composite laminate (43 cm long, 16.5 cm tall and 3 mm thick) for aeronautical use. The panel, subject to axial compression, was not reinforced by any stiffener. According to the manufacturer, the laminate included 1/3 of the layers oriented at 0° (i.e., in the axial direction Y), 1/3 oriented at 90° (i.e., in the transverse direction X) and 1/3 oriented at ±45°. The error functional Ω to be minimized depends on seven unknown structural parameters: four elastic constants E_x_, E_y_, G_xy_ and ν_xy_ and three ply orientations θ_0_, θ_90_ and θ_45_ of the laminate (corresponding, respectively, to nominal angles 0°, 90° and ±45°). The target values of elastic constants indicated by the industrial partner involved in the project are very typical for the IM7/977-2 material: E_x_ = 148300 MPa, E_y_ = 7450 MPa, G_xy_ = 4140 MPa and ν_xy_ = 0.01510.

The optimization process entailed by this identification problem attempts to match the fundamental buckling mode shape of the axially compressed composite panel. Mode shape is normalized with respect to the maximum out-of-plane displacement *w*_max_ occurring at the onset of buckling. Hence, the target quantity of the optimization process is the normalized out-of-plane displacement *w*_norm_ defined as *w*/*w*_max_.

**Figure 8:**
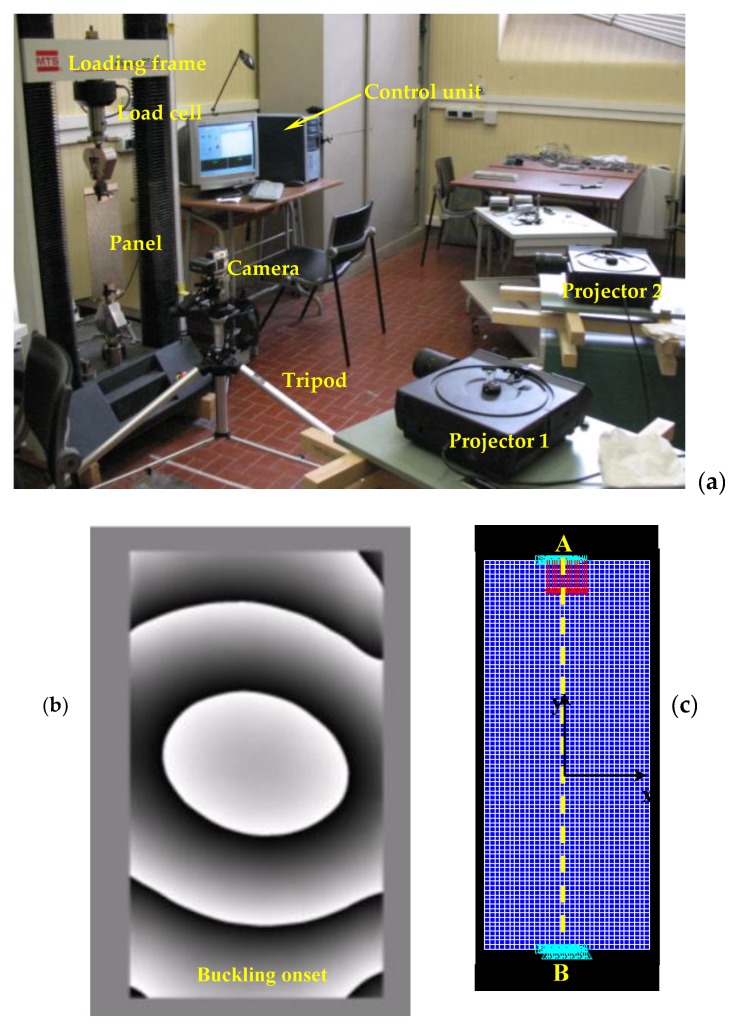
(**a**) Experimental setup used in the identification problem of the axially compressed composite panel; (**b**) Phase pattern at the onset of buckling; (**c**) Finite element model simulating the experiment (control path AB is also indicated).

Figure 8a shows the experimental set-up used for this test case. The axial load is applied to the specimen by imposing a given end-shortening to the panel top edge while bottom edge is fixed. The figure shows the MTS Alliance^TM^ RT/30 testing machine and the grips that realize loading and constraint conditions. In the experiments, end-shortening was progressively increased to 1.5 mm by moving downwards the testing machine cross-bar.

The buckling shape of the panel was measured with a white light double illumination projection moiré set-up [6,181,182]. The experimental setup included two slide projectors (Kodak Ektalite^®^ 500, USA) and a standard digital camera (CANON^®^ Eos 350, 8 Mpix CMOS sensor, Japan) mounted on a tripod; the optical axis of the camera is orthogonal to the panel surface. The illumination angle θ limited by the optical axis of each projector and the optical axis of the camera (i.e., the angle between the direction of illumination and the viewing direction) is 18° while the nominal pitch of the grating is 317.5 μm (80 lines/inch). It can be seen from the figure that projectors are placed symmetrically about the optical axis of the camera. Each projector projects a system of lines and the wave fronts carrying these lines in the space interfere to form an equivalent grating, which is then modulated by the specimen surface. This condition is equivalent to projecting a grating from infinity. The optical set-up is sensitive to out-of-plane displacements which modulate the projected lines making them curve.

The pitch of the projected grating (p_j_) measured on the reference plane (i.e., the surface of the undeformed panel) was 3623.3 μm with a magnification factor of 10.85. Therefore, the sensitivity of the optical setup, p_j_/2tanθ, was 5575.7 μm. The double illumination, together with the subtraction of the phases of the two systems of lines operated via software, produced a phase distribution on both the reference plane and the observed surface that is equivalent to the case of projection from infinity. The phase pattern corresponding to the onset of buckling is shown in Figure 8b. Image processing was done with the HoloStrain software developed by Sciammarella et al. [183].

The experiment was simulated by a finite element model including 8-node shell elements (Figure 8c): again, the selected mesh size guarantees mesh independent solutions and the correspondence between control nodes and image pixels. An eigenvalue buckling analysis was performed in order to determine the critical load of the panel and the corresponding buckled shape.

The error functional Ω was built by comparing finite element results and experimental data at 86 control points along the AB path sketched in Figure 8c. Such a path was chosen in view of the observed symmetry of buckling mode which resembles a typical Euler mode with one half-wave. The maximum out-of-plane displacement occurs at the center of the panel where the origin of the coordinate system X-Y is placed. The following bounds were taken for material parameters in the optimization process: 10000 ≤ E_x_ ≤ 1,000,000 MPa, 1000 ≤ E_y_ ≤ 1,000,000 MPa, 500 ≤ G_xy_ ≤ 20000 MPa and 0.001 ≤ ν_xy_ ≤ 0.1. Ply orientations were made to vary as follows: −50° ≤ θ_0_ ≤ 50°, 0° ≤ θ_90_ ≤ 91°, 10° ≤ θ_45_ ≤ 70°. Similar to the woven composite laminate problem, the bounds imposed on the unknown structural properties are large enough not to affect results of identification process.

The population size of all HS and BBBC variants considered in this study was set equal to 15, slightly more than two times the number of unknown structural parameters. JAYA also was executed with N_POP_ = 15. Values of Ω corresponding to the best design, worst design and center of mass of the initial population are 0.891, 5.149 and 2.303, respectively. The corresponding maximum deviations from target elastic properties are 130.8%, 521% and 730.6%, respectively. Similar to the previous test problem, HFSA, ANSYS and MATLAB-SQP optimization runs were started from the best and worst points as well as from the center of mass of the initial population generated for HS, BBBC and JAYA. Thirty independent optimization runs (keeping N_POP_ = 15) were carried out to statistically evaluate algorithms’ performance.

Table 2 presents the results obtained for this inverse problem. The “SA-Grad” notation now refers to the SA algorithm of Refs. [83,84], which evaluated gradients of error functional via finite differences and used up to [(2·NMP + 1) + 2·NMP] descent directions − out of a total of (2^NMP^ − 1) + 2·NMP potentially available directions—per iteration to form new trial designs. It can be seen that structural properties were identified more accurately by HFSA, HFHS and HFBBBC, that obtained an average error on properties ranging between 0.197 (HFSA) and 0.266% (HFBBBC). The maximum residual error on buckling mode shape evaluated for the identified structural properties never exceeded 2.75% for the present algorithms while was about 3.5% for SA/HS/BBBC-NGR and SA-Grad. Average error on mode shape was always lower than 1.9% for the present algorithms vs. about 2.1% for the other algorithms. The largest errors on *w*-displacements were localized near control path boundaries A and B, that is where displacements tend to zero and numerical noises may occur.

HFHS and HFBBBC were again faster than HFSA as they required, respectively, 478 and 431 structural analyses to complete the optimization process vs. 596 analyses required by HFSA. The proposed algorithms were between 23% and 36% faster than SA/HS/BBBC-NGR variants and even up to 117% faster than SA-Grad. Furthermore, they required much less explosions and 1-D local annealing searches. The fact that HFHS, hybrid HFBBBC and HFSA could reduce the number of finite element analyses with respect to HS/BBBC/SA-NGR and SA-Grad more significantly than for the woven composite problem confirms that increasing the number of design variables may allow the present algorithms to generate more descent directions and speed up the optimization search. Interestingly, the number of structural analyses required in the axially compressed panel identification problem was on average about two times as large as that required in the woven composite laminate identification problem, hence close to the ratio 7 to 4 existing between unknown parameters.

Statistical dispersion on identified properties, residual error on displacements and required number of FE analyses evaluated over the thirty independent runs remained below 0.17% thus confirming the robustness of HFSA, HFHS and HFBBBC. For the sake of brevity, Table 2 does not report the results obtained for AHS [170,171], BBBC-UBS [172], JAYA [35,175,176], MATLAB-SQP and ANSYS also for this problem. MATLAB-SQP and ANSYS converged to solutions (respectively, E_x_ = 148960 MPa; E_y_ = 7594 MPa; G_xy_ = 4189 MPa; ν_xy_ = 0.01488; θ_0_ = 0.07150°; θ_90_ = 86.328; θ_45_ = 43.931° and E_x_ = 146550 MPa; E_y_ = 7255 MPa; G_xy_ = 4146 MPa; ν_xy_ = 0.01558; θ_0_ = 0.1205°; θ_90_ = 88.172; θ_45_ = 41.681°) that still have, respectively, 4.1% and 7.4% errors on ply orientations. This happened after about 600 structural analyses, hence for a higher computational cost than that required by HFSA, HFH and HFBBBC.

As expected, adaptive HS [170,171] and BBBC-UBS [172] were outperformed by HFSA, HFHS and HFBBBC. In particular, adaptive HS converged to the solution E_x_ = 146760 MPa; E_y_ = 7266 MPa; G_xy_ = 4092 MPa; ν_xy_ = 0.01534; θ_0_ = 0.08884°; θ_90_ = 87.632°; θ_45_ = 45.808° after about 1650 structural analyses, about 3.5 times slower than HFHS. Furthermore, BBBC-UBS found the solution E_x_ = 147275 MPa; E_y_ = 7562 MPa; G_xy_ = 3996 MPa; ν_xy_ = 0.01540; θ_0_ = 0.03891°; θ_90_ = 88.513°; θ_45_ = 46.002° after about 1250 structural analyses, about three times slower than HFBBBC. In spite of such a large computational cost, residual errors on structural properties still ranged between 2.6 and 3.5%.

JAYA [35,175,176] converged to the following solution: E_x_ = 149703 MPa; E_y_ = 7451 MPa; G_xy_ = 4999 MPa; ν_xy_ = 0.01525; θ_0_ = 0.01586°; θ_90_ = 85.102°; θ_45_ = 46.171° after 65 iterations and 975 structural analyses. Elastic moduli and Poisson’s ratio were identified very precisely (less than 1% error) but the largest error on layup angles was about 5.8%.

**Figure 9:**
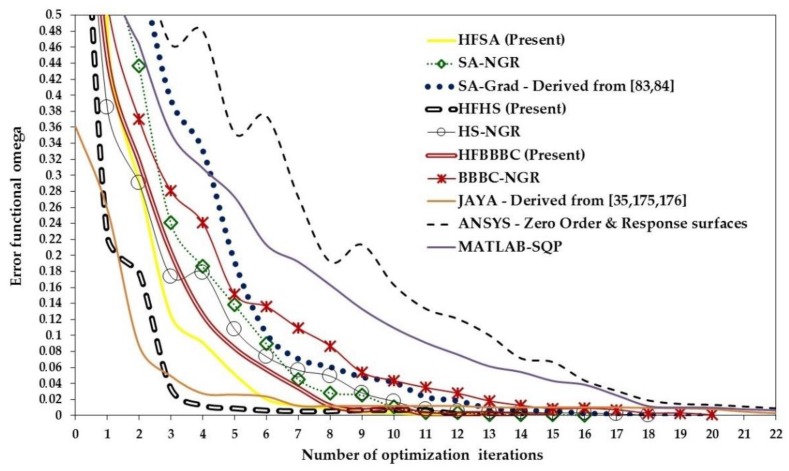
Comparison of convergence behavior of different optimizers in the axially compressed panel identification problem.

The convergence curves relative to the best optimization runs of SA/HS/BBBC variants, JAYA, ANSYS and MATLAB-SQP optimizers are compared in Figure 9. The present algorithms were definitely faster than SA/HS/BBBC-NGR and SA-Grad. In particular, HFSA and HFHS reduced significantly the error functional in the very first iterations but then conducted a fairly long exploitation phase. HFBBBC showed the most regular rate of reduction of Ω and finally required the lowest number of structural analyses overall. Although HFSA optimization was started from an initial design corresponding to a much higher value of Ω than the best design included in the initial population of HFHS and HFBBBC (i.e., Ω = 2.303 vs. Ω = 0.891), it immediately recovered the gap in cost function and the optimization histories of the present SA/HS/BBBC variants became very similar after about 12 iterations. Similar to the previous test problem, JAYA’s best optimization run started from a better population than those generated for the other algorithms: in fact, the best value of error functional was only 0.361. HFHS, HSFA and HFBBBC recovered the initial gap from JAYA within only 3, 6 and 8 iterations, respectively. Furthermore, after the 7th iteration, JAYA’s solutions improved slowly. The higher computational complexity of this test case made hence more evident the inherent limitation of JAYA’s formulation: the absence of a mechanism for directly defining descent directions.

MATLAB-SQP and ANSYS had the slowest converge rate with a marked oscillatory behavior in the latter case. The response surface approximation strategy implemented by ANSYS to build sub-problems (this includes a random selection of the response surface base points) was more efficient than the first-order method used in the woven composite laminate problem. However, it suffered from the noise introduced in the response surface fitting by the very different scales of ply orientations and Poisson’s ratio with respect to elastic moduli.

### 5.4. Bovine Pericardium Patch

The last identification problem solved in this study regarded the mechanical characterization of a glutaraldehyde treated bovine pericardium (GTBP) patch subject to inflation. The intensive experimental campaign conducted in [83,84] confirmed the indications of the industrial partner involved in the project that the GTBP patch behaves as a transversely isotropic hyperelastic material. The same conclusion was achieved from both in-plane equibiaxial tension [83] and 3D inflation [84] tests. Since the bovine pericardium patch can be considered a fibrous hyperplastic material, the error functional Ω to be minimized depends on 17 unknown material parameters: 16 hyperelastic constants (a_1_, a_2_, a_3_ and b_1_, b_2_, b_3_ for the “isotropic” deviatoric term associated to matrix properties; c_2_, c_3_, c_4_, c_5_, c_6_ and d_2_, d_3_, d_4_, d_5_, d_6_ for the “anisotropic” deviatoric term associated to fiber properties) and the fiber orientation direction cosine cosθ. More details on the transversely isotropic hyperelastic constitutive model are given in [174].

The average values of material parameters and their corresponding standard deviations found in [83,84] were: a_1_ = 199.255 ± 0.111, a_2_ = 126.110 ± 0.341, a_3_ = 135.758 ± 1.216, b_1_ = 388.077 ± 1.978, b_2_ = 169.234 ± 2.276, b_3_ = 187.116 ± 1.704, c_2_ = 197.506 ± 1.750, c_3_ = 89.359 ± 0.633, c_4_ = 174.382 ± 0.732, c_5_ = 169.645 ± 0.115, c_6_ = 148.225 ± 0.936, d_2_ = 158.541 ± 0.629, d_3_ = 21.608 ± 0.514, d_4_ = 69.229 ± 0.508, d_5_ = 168.032 ± 2.462, d_6_ = 102.076 ± 0.302 kPa and cosθ = 0.6837 ± 0.000751. Since standard deviations are very small, average values of material properties can be taken as the target result of the identification process. A further proof of the validity of the assumption made above is that cosθ = 0.6837 matches well with the angle of rotation of the iso-displacement contours seen experimentally with respect to the coordinate axes X and Y.

**Figure 10:**
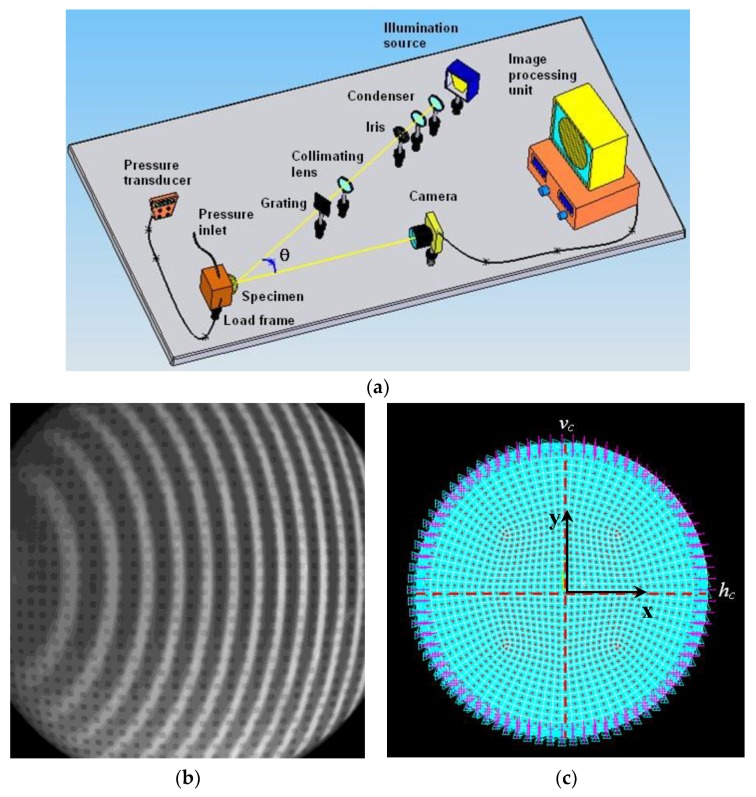
(**a**) Experimental setup used in the identification problem of the bovine pericardium patch; (**b**) Modulation of printed and projected gratings from specimen deformation; (**c**) Finite element model simulating the experiments (control paths *h*_c_ and *v*_c_ are also indicated).

The optimization process entailed by the identification problem attempts to match the total displacements *u*_tot_ = $\sqrt{u^{2}+v^{2}+w^{2}}$ of a circular membrane of diameter 40 mm and thickness 0.5 mm progressively inflated up to the maximum pressure of 12.22 kPa. An assembly view of the experimental set-up used in the inflation test is shown in Figure 10a. 3D displacement components were simultaneously measured by combining intrinsic moiré (IM) and projection moiré (PM) [5,6,7]: IM is sensitive to in-plane displacements while PM is sensitive to out-of-plane displacements.

Figure 10b shows a typical image of the inflated membrane illuminated by white light with the two modulated gratings by the deformed specimen: the printed square-dots grating (1 mm pitch) follows the evolution of the in-plane displacements *u* and *v* while the projected vertical lines grating (2 mm pitch) follows the evolution of the out-of-plane displacement *w*. The origin of the reference system X-Y is put in the center of the tested membrane. Images were processed with the HoloStrain software [181]. The largest in-plane displacement measured in the experiments was about 0.5 mm while the largest out-of-plane displacement was about 5.3 mm. Each displacement component was extracted from the FFT pattern of the recorded image by properly selecting spatial frequencies. More details on the experimental tests performed for this identification problem are given in [83,84].

The inflation test was simulated by the finite element model shown in Figure 10c, including 1200 quadratic solid hyperelastic elements and 8603 nodes. The FE model shows the zero-displacement boundary condition imposed at the circular edge of the membrane as well as the uniformly distributed inflation pressure acting on the membrane. Mesh size was determined via convergence analysis again taking care to match control nodes and pixels of the recorded images. In the FE analysis, the NLGEOM geometric nonlinearity option was activated in order to account for the large deformations experienced by the hyperelastic membrane.

The error functional Ω of this test problem was built by comparing ANSYS results and moiré data at 81 control points located on the horizontal control path *h*_c_ along the X-axis and the vertical control path *v*_c_ along the Y-axis. All hyperelastic constants were made to vary between 10 kPa and 1 MPa while cosθ could range between 0.65 and 0.75. As for the previous two test problems, the bounds imposed on material properties were large enough not to affect the solution of the identification process.

The population size of all HS and BBBC variants considered in this study was set equal to 30, hence about two times the number of unknown material parameters. JAYA’s optimization also was run with N_POP_ = 30. The values of Ω functional corresponding to the best design, worst design and center of mass of the initial population are 0.125, 0.570 and 0.340, respectively. The corresponding average deviation from target properties ranges between 223% and 566%. The high nonlinearity of this identification problem is confirmed by the fact that the best four designs of the population present a larger deviation from target properties than the worst design: respectively, 417%, 345%, 350% and 432% vs. 282%. HFSA, ANSYS and MATLAB-SQP optimization runs were started from the best and worst points as well as from the center of mass of the initial population generated for HS and BBBC. Thirty independent runs were executed with different initial populations (keeping N_POP_ = 30) to statistically evaluate performance of different optimizers.

Table 3 presents the results obtained for this inverse problem. The “SA-Grad” notation again refers to the SA algorithm derived from [83,84]. The present SA/HS/BBBC variants were once again more accurate than SA/HS/BBBC-NGR algorithms. In fact, average and maximum errors on identified material properties, respectively, ranged between 0.196 (HFHS) and 0.256% (HFSA), and between 0.594% (HFBBBC) and 0.692% (HFHS) vs. about 0.49% (average) and 1.24% (maximum) errors seen for SA/HS/BBBC-NGR. The largest residual error on *u*_tot_-displacements evaluated for the present algorithms never exceed 2.9% and average residual errors were about 30% lower than for SA/HS/BBBC-NGR. The analysis of error maps revealed that largest errors are localized at X = ± 11.5 mm and Y = ± 8.5 mm, that is where the three displacement components become comparable in magnitude.

HFHS, HFBBBC and HFSA required, respectively, 1223, 1294 and 1373 structural analyses to complete the optimization process. Hence, the proposed algorithms were between 22.4% and 32.3% faster than the other algorithms and reduced the number of explosions and 1-D local annealing searches on average by a factor 5 with respect to the previous formulations. The average number of FE analyses required by HFSA, HFHS and HFBBBC for this identification problem was about 2.5 times as large as its counterpart in the axially compressed panel problem. Hence, computational cost again changed almost linearly with the number of unknown parameters.

The HFSA, HFHS and HFBBBC algorithms were very robust also for this test problem. In fact, statistical dispersion on identified properties, residual error on displacements and required number of FE analyses evaluated over the thirty independent runs remained below 0.058%. The higher amount of design freedom introduced by the larger number of unknown parameters with respect to the first two test problems allowed to generate high quality trial solutions more easily regardless of the composition of the initial population.

AHS [170,171], BBBC-UBS [172], MATLAB-SQP and ANSYS were outperformed by HFSA, HFHS and HFBBBC also in this identification problem. In particular, the MATLAB-SQP solution (a_1_ = 196.6, a_2_ = 126.8, a_3_ = 130.58, b_1_ = 392.17, b_2_ = 169.72, b_3_ = 186.97, c_2_ = 196.52, c_3_ = 89.768, c_4_ = 173.46, c_5_ = 168.98, c_6_ = 148.79, d_2_ = 159.12, d_3_ = 21.173, d_4_ = 69.507, d_5_ = 165.86, d_6_ = 98.219 kPa and cosθ = 0.6882) was obtained after about 80 optimization iterations and 1550 structural analyses (i.e., 1.13 times the computational cost of HFSA) but still had a 3.8% error on hyperelastic constants a_3_ and d_6_. ANSYS converged to a slightly worse solution than MATLAB-SQP even though it required about 100 optimization iterations and 1700 finite element analyses.

BBBC-UBS [172] converged to the solution a_1_ = 199.368, a_2_ = 123.056, a_3_ = 135.963, b_1_=386.842, b_2_ = 170.251, b_3_ = 186.915, c_2_ = 198.753, c_3_ = 89.669, c_4_=175.105, c_5_ = 169.838, c_6_ = 147.961, d_2_ = 158.403, d_3_ = 21.681, d_4_ = 69.146, d_5_ = 166.405, d_6_ = 102.738 kPa and cosθ = 0.6833 after about 2800 finite element analyses, about 2.2 times slower than HFBBBC. However, the residual error made on the a_2_ hyperelastic constant was still 2.4% in spite of such a large computational cost. Adaptive HS [170,171] was again the worst optimizer overall as its solution (a_1_ = 197.213, a_2_ = 125.214, a_3_ = 130.744, b_1_ = 388.321, b_2_ = 174.278, b_3_ = 186.831, c_2_ = 197.451, c_3_ = 88.118, c_4_ = 172.409, c_5_ = 168.746, c_6_ = 151.330, d_2_ = 158.462, d_3_ = 20.845, d_4_ = 69.298, d_5_ = 168.764, d_6_ = 100.741 kPa and cosθ = 0.6776) yield a residual error of 3.5% on hyperelastic constants a_3_ and d_3_, in spite of having performed about 3500 finite element analyses, about 2.9 times more than HFHS.

JAYA [35,175,176] obtained a very close solution to BBBC-UBS [172] (i.e., less than 0.25% difference on hyperelastic constants; same value of cosθ) completing the optimization process in 2380 finite element analyses (i.e., 140 iterations), still very slowly with respect to the present SA/HS/BBBC variants. The detail of the JAYA’s solution is as follows: a_1_ = 199.325, a_2_ = 122.987, a_3_ = 136.094, b_1_ = 386.915, b_2_ = 170.351, b_3_ = 187.240, c_2_ = 198.259, c_3_ = 89.655, c_4_ = 174.948, c_5_ = 170.087, c_6_ = 148.022, d_2_ = 158.558, d_3_ = 21.634, d_4_ = 69.093, d_5_ = 166.083, d_6_ = 102.753 kPa and cosθ = 0.6833.

**Figure 11:**
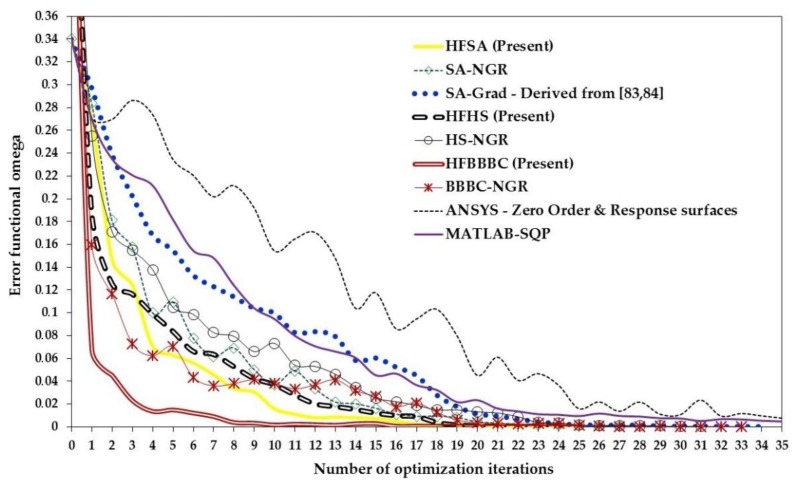
Comparison of convergence behavior of different optimizers in the bovine pericardium patch identification problem.

The convergence curves obtained for the best optimization runs of HS/BBBC/SA variants, MATLAB-SQP and ANSYS optimizers are compared in Figure 11. The JAYA’s best run curve is not shown in the figure as values of Ω recorded in the first 30 iterations are off-scale. Because of the high nonlinearity of this problem, intermediate designs were sorted also in terms of deviation from target properties. This explains why convergence curves of BBBC and HS variants start from higher values of Ω than those of SA variants, ANSYS and SQP optimizers which start from the Ω = 0.340 value corresponding to the center of mass of the population. HFBBBC was definitely the fastest algorithm throughout optimization process, followed by HFSA and HFHS. However, convergence curve of HFHS was almost monotonic and this explains why HFHS finally required less finite element analyses than HFBBBC and HFSA, which instead showed fairly long steps with small improvements in solution. The optimization histories of HFSA, HFHS and HFBBBC practically coincided after 20 iterations.

The present algorithms were definitely faster than SA/HS/BBBC-NGR variants and SA-Grad. The “NGR” algorithms showed steps and oscillatory behavior in the optimization history because the number of descent directions (i.e., one or two) involved in the formation of new trial solutions was not large enough to deal with the high nonlinearity of the GTBP patch identification problem. Interestingly, SA-Grad considered a set of 69 (i.e., 4·NMP + 1) descent directions in each iteration to update design. This allowed oscillatory behavior to be limited but about one half of these 69 directions were formed by perturbing one material parameter at a time and hence yield little improvements in the error functional.

ANSYS again showed the slowest converge rate and a marked oscillatory behavior. That happened because the high nonlinearity of this identification problem reduced the efficiency of the response surface approach used by ANSYS for building the approximate sub-problem in each optimization iteration. MATLAB-SQP was competitive with SA-Grad for about 20 iterations but it then started to cycle between intermediate designs characterized by Ω = 0.01 trying to find proper values for hyperelastic constants a_3_ and d_6_. Conversely, SA-Grad used its inherent exploitation capability to improve solution in the final part of optimization history.

JAYA (the convergence curve is not shown in the figure) started its best optimization run from a population with Ω _OPT_ = 0.476 and could not reduce the error functional value below 0.35 for the first 30 iterations. Such a behavior confirms that as problem size increases it becomes more important to update population according to the rank held in the population by the currently perturbed design. This requirement is certainly satisfied by HFHS and HFBBBC, which generate search directions **S_FAST_**, **S_BEST_**, **S_2ndBEST_**, **S_OPT−CM_** etc. (or perform low cost evaluations of sensitivities of error functional) while JAYA simply perturbs designs following the initial order assigned to the N_POP_ individuals. For a given design, it is also important to tailor perturbations of each variable to sensitivities of error functional. Whilst this is intrinsically done by 1D probabilistic search utilized by the present algorithms, JAYA updates variables just following the classical 1st to NMPth variable sequence. The latter may result in missing “good” values of “some” variable that potentially improve a given solution more than other values of other variables selected instead. The probability of missing good variable values clearly increases with the problem dimension as it becomes more difficult to reconstruct in the search space the path leading to the global optimum. This explains the “inertia” effect observed for JAYA, which became slower as test problem size increased. Since the ratio between population and number of optimization variables was very similar for all test cases, the “inertia” effect logically occurred also for larger population sizes (see discussion on JAYA’s results developed in Section 5.1).

In order to evaluate sensitivity of convergence behavior of HFSA, HFHS and HFBBBC algorithms to initial population and initial design, the bovine pericardium patch identification problem was also solved with a population including 90 candidate solutions. The new population of HS and BBBC included 60 additional “low quality” candidate solutions characterized by higher values of the error functional: the new worst design has Ω = 1.352 while the new center of mass has Ω = 0.709. Consequently, the average deviation from target properties varied between 223% and 1030%. The candidate solutions yielding the lowest values of Ω again did not show the smallest deviations from target properties.

**Figure 12:**
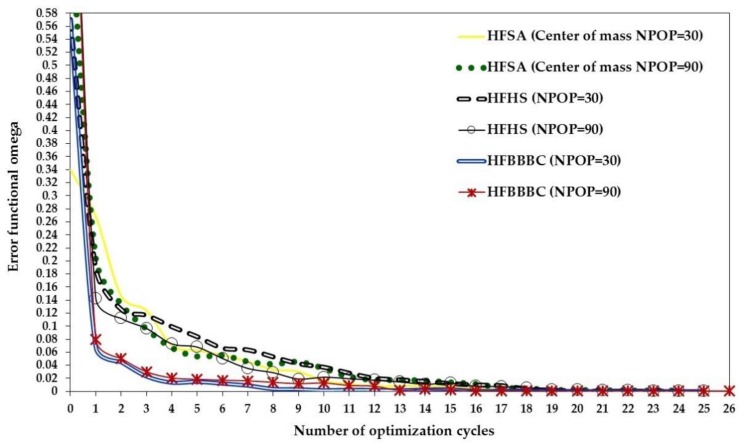
Sensitivity of HFSA, HFHS and HFBBBC to initial population/starting point for the bovine pericardium patch identification problem.

Results of sensitivity analysis to population size and initial solutions are presented in Table 4. All of the present algorithms practically converged to the same material properties regardless of population size/initial design. Deviations from target material properties were slightly higher for N_POP_ = 90 but remained below 0.27% (average error) and 0.78% (maximum error). Residual errors on displacements also changed marginally with respect to those evaluated for N_POP_ = 30. HFHS and HFBBBC performed one more iteration than in the case N_POP_ = 30 while HFSA could eliminate one optimization cycle. The number of explosions and 1-D local annealing searches remained the same for the two populations. The number of finite element analyses required in the optimization process changed at most by 15%. This increase was due to the fact that the 90-designs population included lower quality solutions. The robustness of the present algorithms is confirmed by the convergence curves shown in Figure 12. It appears that each pair of optimization histories relative to a given algorithm practically coincided in the last 4–5 iterations.

## 6. Discussion and Conclusions

This study presented a hybrid framework for mechanical identification of materials and structures. The framework combined full-field measurements done with optical methods and global optimization based on metaheuristic algorithms. Such a choice was motivated by the fact that metaheuristic algorithms allow to efficiently deal with the inherent non-linearity and non-convexity of inverse problems. From the experimental point of view, using optical methods is the best approach to identification problems because these techniques provide full-field information, do not alter the state of the investigated specimen, and can precisely detect material anisotropy, presence of local defects and/or damage.

However, the “no free lunch” theorem states that no metaheuristic algorithm can outperform all other algorithms in all optimization problems. Unlike gradient-based optimizers that are still implemented in commercial software although their formulations did not change much in the last 25–30 years, most of the newly developed metaheuristic algorithms added very little to the optimization practice and their appeal quickly vanished after a very few years. This suggests that rather than proposing a new metaheuristic algorithm that improves available methods just marginally it is better to significantly improve the most powerful algorithms. For this reason, we developed three advanced versions of simulated annealing (SA), harmony search (HS) and big bang-big crunch (BBBC). These algorithms were selected as they are very well established metaheuristic optimization methods for which many successful applications to inverse problems have been documented in technical literature. Furthermore, these algorithms possess important features, which are very desirable in global optimization. In fact, SA is inherently able to bypass local optima, HS has a memory where good solutions may be stored, BBBC employs the concept of center of mass including information on the average quality of the population of trial solutions.

The rationale behind the new algorithms developed in this study—denoted as Hybrid Fast Simulated Annealing (HFSA), Hybrid Fast Harmony Search (HFHS) and Hybrid Fast Big Bang-Big Crunch (HFBBBC)—was to generate high quality trial designs lying on a properly selected set of descent directions. For that purpose, enhanced approximate line search and computationally cheap gradient evaluation strategies were developed. Besides hybridizing SA/HS/BBBC metaheuristic search engines with gradient information and approximate line search, HS and BBBC were hybridized with an enhanced 1-D probabilistic search derived from SA.

The optimization framework was tested in three inverse elasticity problems: (i) mechanical characterization of a composite laminate used as substrate in electronic boards; (ii) mechanical characterization and layup identification of an axially compressed composite panel for aeronautical use; (iii) mechanical characterization of bovine pericardium patches used in biomedical applications. The largest test case (iii) included 17 unknown parameters. Sensitivity of inverse problem solutions and convergence behavior to population size and initial design/population was statistically evaluated. A preliminary mathematical optimization problem was solved in order to train algorithms. Remarkably, HFSA, HFHS and HFBBBC were very efficient and outperformed other SA, HS and BBBC formulations, the JAYA algorithm as well as a state-of-the-art gradient-based optimizer like MATLAB-SQP. Furthermore, the present algorithms always were very robust.

An interesting fact observed from Table 1, Table 2, Table 3 and Table 4 is that the maximum residual error on displacements was about 3% for all identification problems. In order to check if this is an inherent limitation of HFSA, HFHS and HFBBBC algorithms, an *in silico* identification was carried out by computing displacement fields for the target material/structural properties provided by manufacturers (first two identification problems) or determined by averaging results of [83,84] (last identification problem). Optimizations were hence run to reconstruct the target displacement fields generated numerically. Remarkably, the present algorithms were always able to converge to the target material/structural properties reproducing the displacement field with zero residual errors. The observed 3% maximum error hence falls within the level of uncertainty normally entailed by a FEMU-based characterization process, a very complicated task which attempts to match experimental data and finite element results. Optically measured displacements certainly are a good target because the accuracy of speckle and moiré techniques may go down to a very small fraction of the sensitivity of experimental setup. However, in this study, control points were selected right at critical locations (i.e., near boundaries where displacements tend to zero or in transition regions where all displacement components are comparable in magnitude) where even small drifts on measured quantities may have an impact on the success of the identification process. Furthermore, a rather small set of control points was selected for building the error functional Ω. This was done in order to make the matching of experimental data and numerical results more difficult, thus testing the real ability of HFSA, HFHS and HFBBBC to find the global optimum or get very close to the global optimum. In view of this, the results presented in this article should be considered very satisfactory.

Based on the arguments discussed above, it can be concluded that the proposed hybrid framework is a powerful tool for solving inverse mechanical problems.

## Figures and Tables

**Table 1:** Results of the identification process carried out for the woven composite laminate.

Material Properties	HFSA [Present]	SA-NGR	SA-Grad [77]	HFHS [Present]	HS-NGR	HFBBBC [Present]	BBBC-NGR
E_x_	24953	24838	25043	25034	25074	25047	25051
E_y_	22051	22207	22034	21995	21913	21978	21861
ν_xy_	0.280	0.278	0.279	0.279	0.279	0.279	0.279
G_xy_	5008	5010	5000	5005	5017	5003	4994
Error on material properties (%)	Aver: 0.145 Max: 0.232	Aver: 0.626 Max: 0.941	Aver: 0.171 Max: 0.357	Aver: 0.154 Max: 0.357	Aver: 0.347 Max: 0.395	Aver: 0.176 Max: 0.357	Aver: 0.328 Max: 0.632
Residual error on displacements (%)	Aver: 0.545 Max: 2.532	Aver: 0.591 Max: 2.788	Aver: 0.643 Max: 2.736	Aver: 0.580 Max: 2.689	Aver: 0.622 Max: 2.903	Aver: 0.640 Max: 2.734	Aver: 0.626 Max: 2.899
Optimization iterations	14 (1-D SA = 1)	17 (1-D SA = 5)	22 (1-D SA = 10)	13	15	12 (N_exp_ = 1)	13 (N_exp_ = 4)
FE analyses	257	332	388	210	242	222	274

**Table 2:** Results of the identification process carried out for the axially compressed composite panel.

Material Properties	HFSA [Present]	SA-NGR	SA-Grad [77]	HFHS [Present]	HS-NGR	HFBBBC [Present]	BBBC-NGR
E_x_	148708	149626	148730	147928	146831	148343	147406
E_y_	7479	7403	7516	7491	7464	7499	7523
ν_xy_	0.01508	0.01502	0.01520	0.01510	0.01527	0.01509	0.01517
G_xy_	4140	4107	4107	4145	4160	4160	4163
θ_0_	0.001021	0.001022	0.0009930	0.003492	0.003599	0.003243	0.0009077
θ_90_	90.223	89.347	90.800	89.904	89.720	90.274	90.685
θ_45_	44.938	45.055	44.975	45.113	45.359	44.975	44.905
Error (%) on parameters	Aver: 0.197 Max: 0.389	Aver: 0.617 Max: 0.894	Aver: 0.597 Max: 0.889	Aver: 0.213 Max: 0.550	Aver: 0.649 Max: 1.126	Aver: 0.266 Max: 0.658	Aver: 0.596 Max: 0.980
Error on mode shape (%)	Aver: 1.554 Max: 2.281	Aver: 2.088 Max: 3.453	Aver: 2.107 Max: 3.580	Aver: 1.680 Max: 2.504	Aver: 2.072 Max: 3.426	Aver: 1.889 Max: 2.735	Aver: 2.103 Max: 3.497
Optimization iterations	13 (1-D SA = 2)	16 (1-D SA = 5)	20 (1-D SA = 6)	14	18	15 (N_exp_ = 2)	20 (N_exp_ = 11)
FE analyses	596	863	935	478	672	431	660

**Table 3:** Results of the identification process carried out for the bovine pericardium patch.

Material Properties	HFSA [Present]	SA-NGR	SA-Grad Derived from [83,84]	HFHS [Present]	HS-NGR	HFBBBC [Present]	BBBC-NGR
a_1_	198.961	198.787	199.255	198.837	200.989	199.246	199.362
a_2_	126.885	127.046	126.110	125.858	127.761	125.361	127.680
a_3_	135.601	134.819	135.758	135.587	135.349	136.194	136.205
b_1_	386.003	385.440	388.077	388.455	390.833	388.284	387.069
b_2_	169.684	170.439	169.234	169.245	169.568	168.627	170.263
b_3_	187.298	185.346	187.116	187.383	188.867	187.568	187.567
c_2_	197.367	197.407	197.506	197.783	197.959	197.896	198.615
c_3_	89.111	89.568	89.359	89.192	89.594	89.064	89.661
c_4_	173.820	173.634	174.382	174.239	174.382	173.938	174.938
c_5_	169.835	171.097	169.645	170.031	170.209	169.607	169.833
c_6_	147.876	149.953	148.225	148.261	147.013	148.060	148.043
d_2_	159.260	159.449	158.541	158.699	158.268	158.595	158.417
d_3_	21.692	21.794	21.608	21.705	21.599	21.525	21.512
d_4_	69.112	69.275	69.229	69.063	69.682	69.041	69.280
d_5_	167.501	166.533	168.032	169.194	168.956	168.035	166.372
d_6_	102.293	102.551	102.076	101.687	101.573	102.410	102.654
cosθ_f_	0.6838	0.6837	0.6837	0.6839	0.6776	0.6837	0.6834
Errors on properties (%)	Aver: 0.256 Max: 0.615	Aver: 0.565 Max: 1.166	N/A	Aver: 0.196 Max: 0.692	Aver: 0.516 Max: 1.309	Aver: 0.206 Max: 0.594	Aver: 0.376 Max: 1.245
Residual errors on *u*_tot_ (%)	Aver: 1.141 Max: 2.845	Aver: 1.666 Max: 3.254	Aver: 1.560 Max: 3.167	Aver: 1.070 Max: 2.903	Aver: 1.533 Max: 3.089	Aver: 1.097 Max: 2.904	Aver: 1.658 Max: 3.226
Optimization iterations	25 (1-D SA = 2)	27 (1-D SA = 8)	34 (1-D SA = 12)	24	31	25 (N_exp_ = 2)	33 (N_exp_ = 9)
FE analyses	1373	1829	1972	1223	1807	1294	1668

**Table 4:** Bovine pericardium patch identification problem: sensitivity of HFHS, HFBBBC and HFSA algorithms to initial design/population.

Material Properties	HFSA (N_POP_ = 30)	HFSA (N_POP_ = 90)	HFHS (N_POP_ = 30)	HFHS (N_POP_ = 90)	HFBBBC (N_POP_ = 30)	HFBBBC (N_POP_ = 90)
a_1_	198.961	198.493	198.837	199.408	199.246	198.978
a_2_	126.885	126.010	125.858	126.093	125.361	125.888
a_3_	135.601	136.301	135.587	135.970	136.194	135.857
b_1_	386.003	387.236	388.455	388.961	388.284	390.563
b_2_	169.684	169.151	169.245	168.943	168.627	168.873
b_3_	187.298	186.850	187.383	186.345	187.568	185.810
c_2_	197.367	197.909	197.783	197.181	197.896	197.948
c_3_	89.111	89.247	89.192	89.809	89.064	89.264
c_4_	173.820	174.274	174.239	174.325	173.938	174.047
c_5_	169.835	169.719	170.031	169.600	169.607	169.458
c_6_	147.876	149.097	148.261	148.307	148.060	148.189
d_2_	159.260	157.591	158.699	157.380	158.595	158.459
d_3_	21.692	21.697	21.705	21.725	21.525	21.714
d_4_	69.112	69.182	69.063	69.354	69.041	69.287
d_5_	167.501	168.849	169.194	168.994	168.035	169.337
d_6_	102.293	102.550	101.687	102.141	102.410	102.204
cosθ_f_	0.6838	0.6851	0.6839	0.6837	0.6837	0.6846
Errors on properties (%)	Aver: 0.256 Max: 0.615	Aver: 0.266 Max: 0.599	Aver: 0.196 Max: 0.692	Aver: 0.231 Max: 0.732	Aver: 0.206 Max: 0.594	Aver: 0.250 Max: 0.777
Residual errors on *u*_tot_ (%)	Aver: 1.141 Max: 2.845	Aver: 1.257 Max: 2.896	Aver: 1.070 Max: 2.903	Aver: 1.188 Max: 2.907	Aver: 1.097 Max: 2.904	Aver: 1.201 Max: 2.962
Optimization iterations	25 (1-D SA = 2)	24 (1-D SA = 2)	24	25	25 (N_exp_ = 2)	26 (N_exp_ = 2)
FE analyses	1373	1396	1223	1414	1294	1377
